# Supplementary material for: Identification of Two Distinct Immune Subtypes in Hepatitis B Virus (HBV)-Associated Hepatocellular Carcinoma (HCC)
Source: Cancers (Basel). 2024 Mar 30;16(7):1370. doi: 10.3390/cancers16071370 (PMC11011136; doi:10.3390/cancers16071370)
Supplement: Supplementary file 1 [file cancers-16-01370-s001.zip › cancers-2857282-supplementary.pdf]

Identification of Two Distinct Immune Subtypes in Hepatitis B Virus (HBV)-Associated Hepatocellular Carcinoma (HCC)

Davide De Battista[1], Rylee Yakymi[1,*], Evangeline Scheibe[1,*], Shinya Sato[1], Hannah Gerstein[1], Tovah E. Markowitz[2], Justin Lack [3], Roberto Mereu[4], Cristina Manieli[5], Fausto Zamboni[4], Patrizia Farci [1,+]

*Equally contributed

[1] Hepatic Pathogenesis Section, Laboratory of Infectious Diseases, National Institute of Allergy and Infectious Diseases, National Institutes of Health, Bethesda, MD 20892

[2] Integrated Data Sciences Section, Research Technologies Branch, National Institute of Allergy and Infectious Diseases, National Institutes of Health, Bethesda, MD 20892, USA

[3] NIAID Collaborative Bioinformatics Resource, National Institute of Allergy and Infectious Diseases, National Institutes of Health, Bethesda, MD 20892, USA

[4] Department of Surgery and Liver Transplantation Center, Azienda Ospedaliera Brotzu, Cagliari, Italy

[5] Sevizio di Anatomia Patologica, Azienda Ospedaliera Brotzu, Cagliari, Italy

[+] Address correspondence to Dr. Patrizia Farci, Hepatic Pathogenesis Section, Laboratory of Infectious Diseases, NIAID, NIH, Bethesda MD, USA; email: patrizia.farci@nih.gov

This file includes:

Figures S1 to S8

Tables S1 to S5

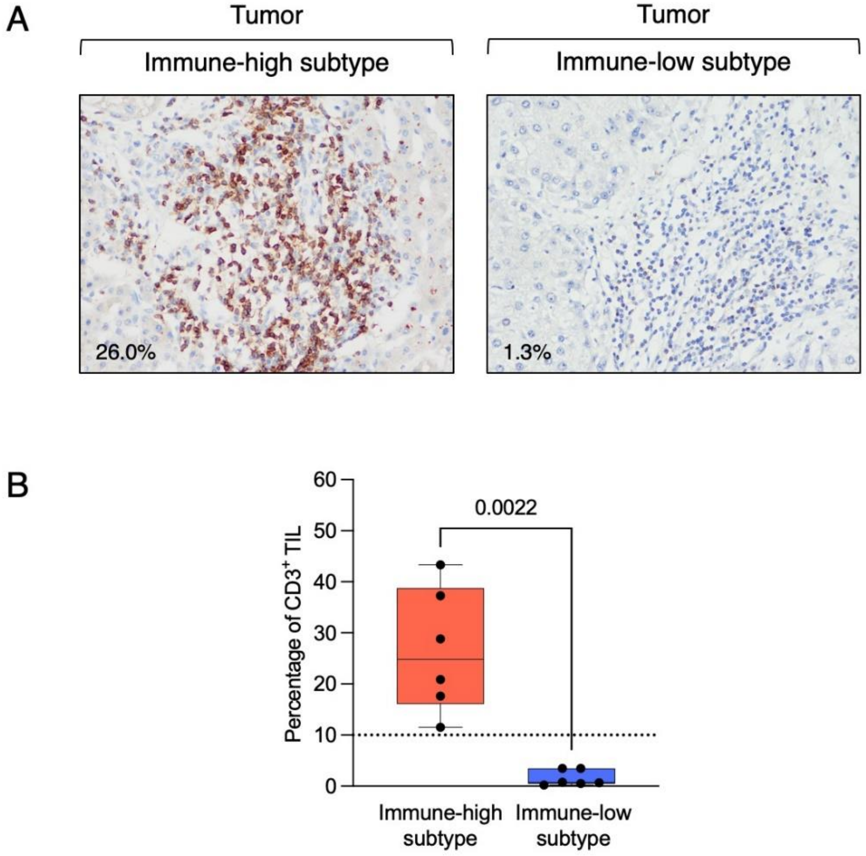

Figure S1. Identification of two distinct immune subtypes in HBV-HCC by IHC. (A) The images illustrate the immunostaining with monoclonal antibodies against CD3 (T cells) in a representative case from the immune-high and the immune-low HCC subtype (200x magnification). The percentage of TIL in the left bottom corner of each image was evaluated according to the guidelines of the international TIL working group. (B) Whisker plots show the percentage of CD3-positive TIL from each patient in the two subtypes. The median value of each subtype is indicated by a horizontal line in each box, with the 25th and 75th percentiles indicated at the top and bottom of each box; the min and max values are indicated at the top and bottom of each I bar. The dotted line indicates the threshold used to define immune-high (percentage of TIL greater than 10%) and immune-low (percentage of TIL lower than 10%) subtype. Statistical significance was determined using the Mann-Whitney test for the comparison between the two subtypes.

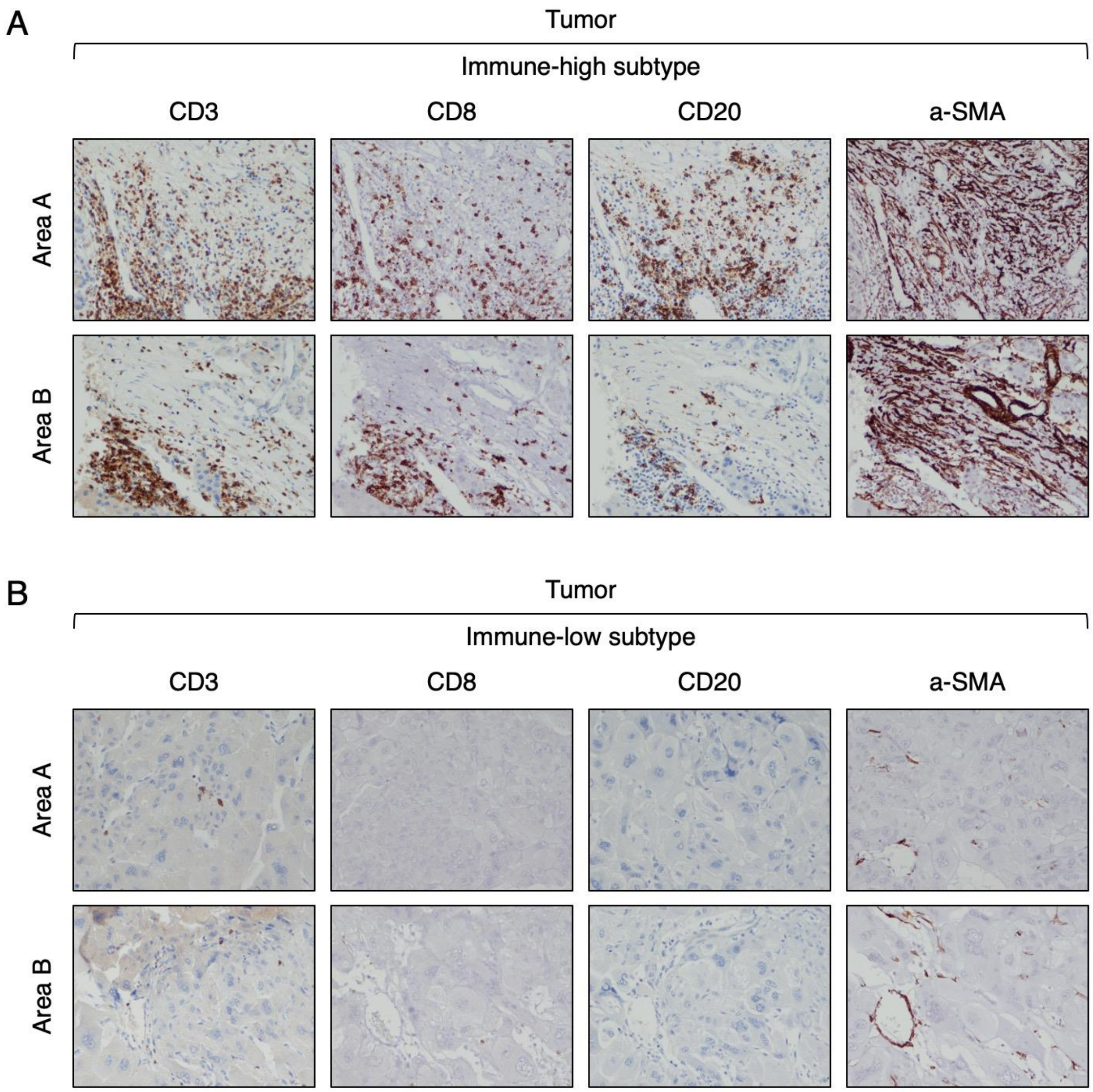

Figure S2. Expression of immune markers in different liver compartments in a representative HCC case from the immune-high (Pt. H4) and the immune-low (Pt. C4) subtypes. The images illustrate the immunostaining with monoclonal antibodies against CD3 (T cells), CD8 (CD8 T cells), CD20 (B cells), and alpha-SMA (stromal activation) in paraffin liver sections from the center (area A) and the periphery of the tumor (area B) in both subtypes (200x magnification).

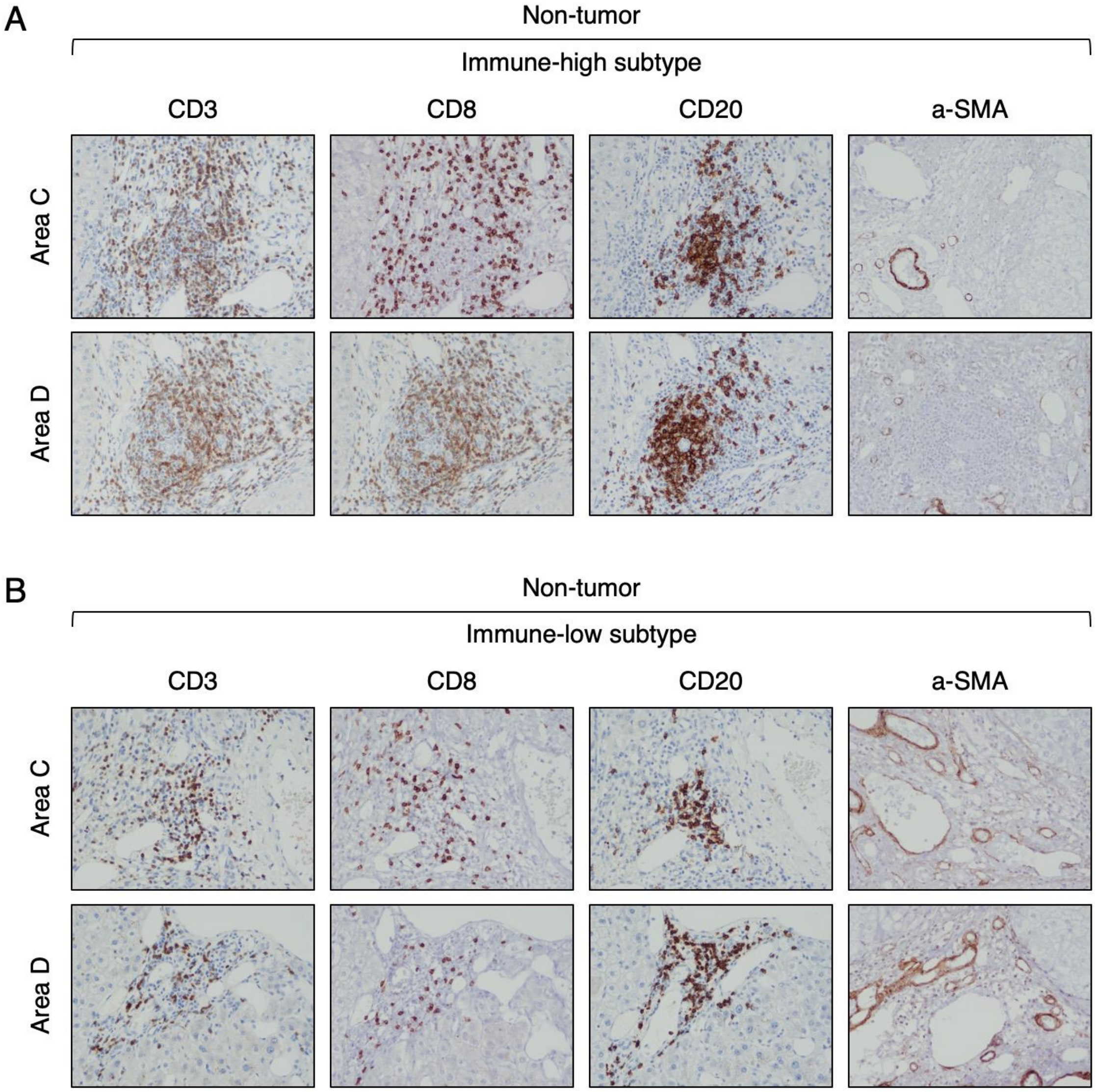

Figure S3. Expression of immune markers in the surrounding nontumorous tissue of a representative case from the immune-high (Pt. H4) and the immune-low (Pt. C4) subtypes. The images illustrate the immunostaining with monoclonal antibodies against CD3 (T cells), CD8 (CD8 T cells), CD20 (B cells), and alpha-SMA (hepatic stellate cells) in paraffin liver sections from the perilesional area (area C) and the most distant area from the center of the tumor (area D) in both subtypes (200x magnification).

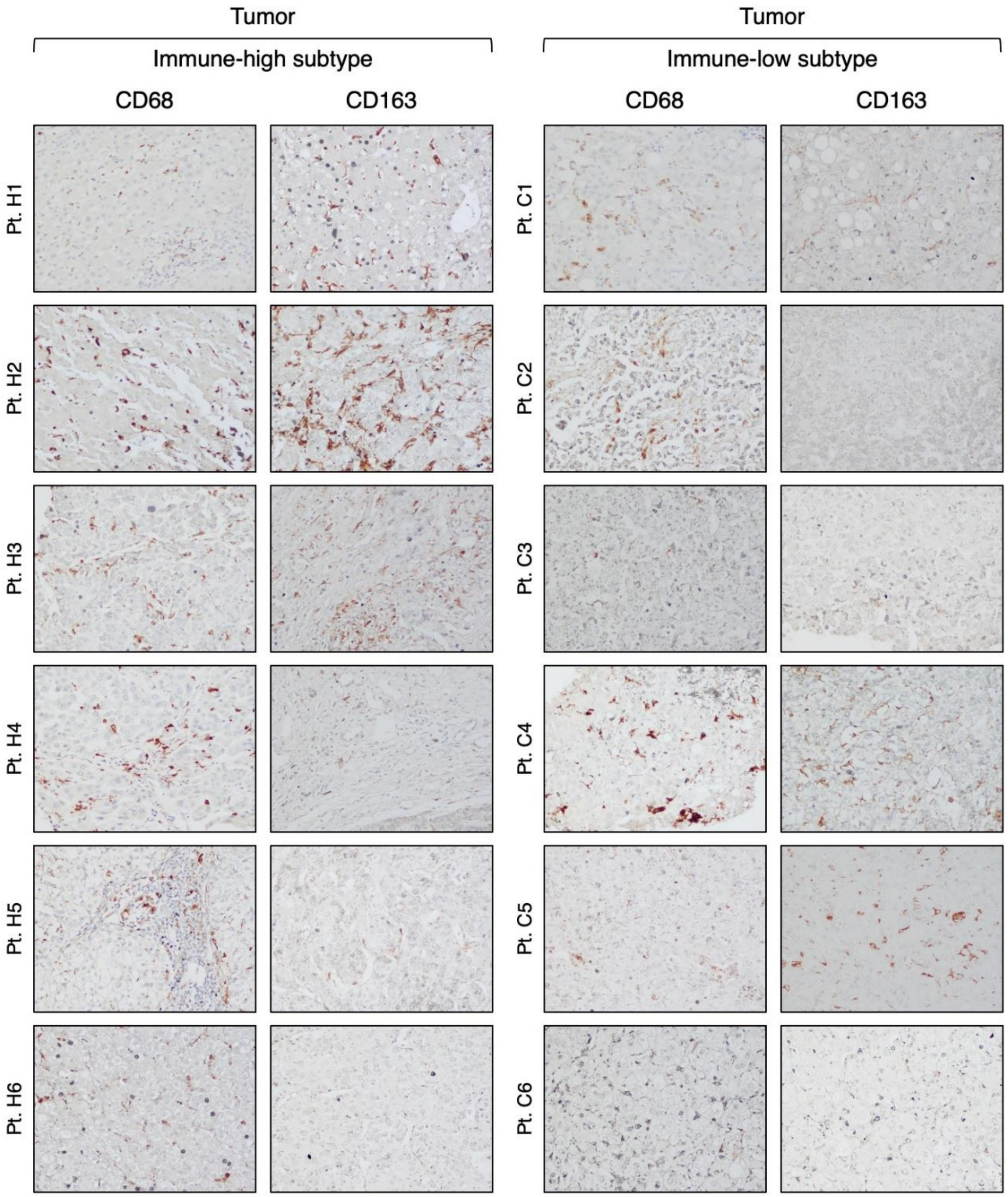

Figure S4. Immunohistochemical staining of CD68 and CD163 markers in the center of the tumor of immune-high and immune-low HCC subtypes. Liver specimens obtained at the time of liver transplantation were stained with monoclonal antibodies against CD68 (monocytes and Kupfer cells) and CD163 (M2-like macrophages). This figure shows all 12 patients with immune-high subtype (left two columns) and immune-low subtype (right two columns) (200x magnification).

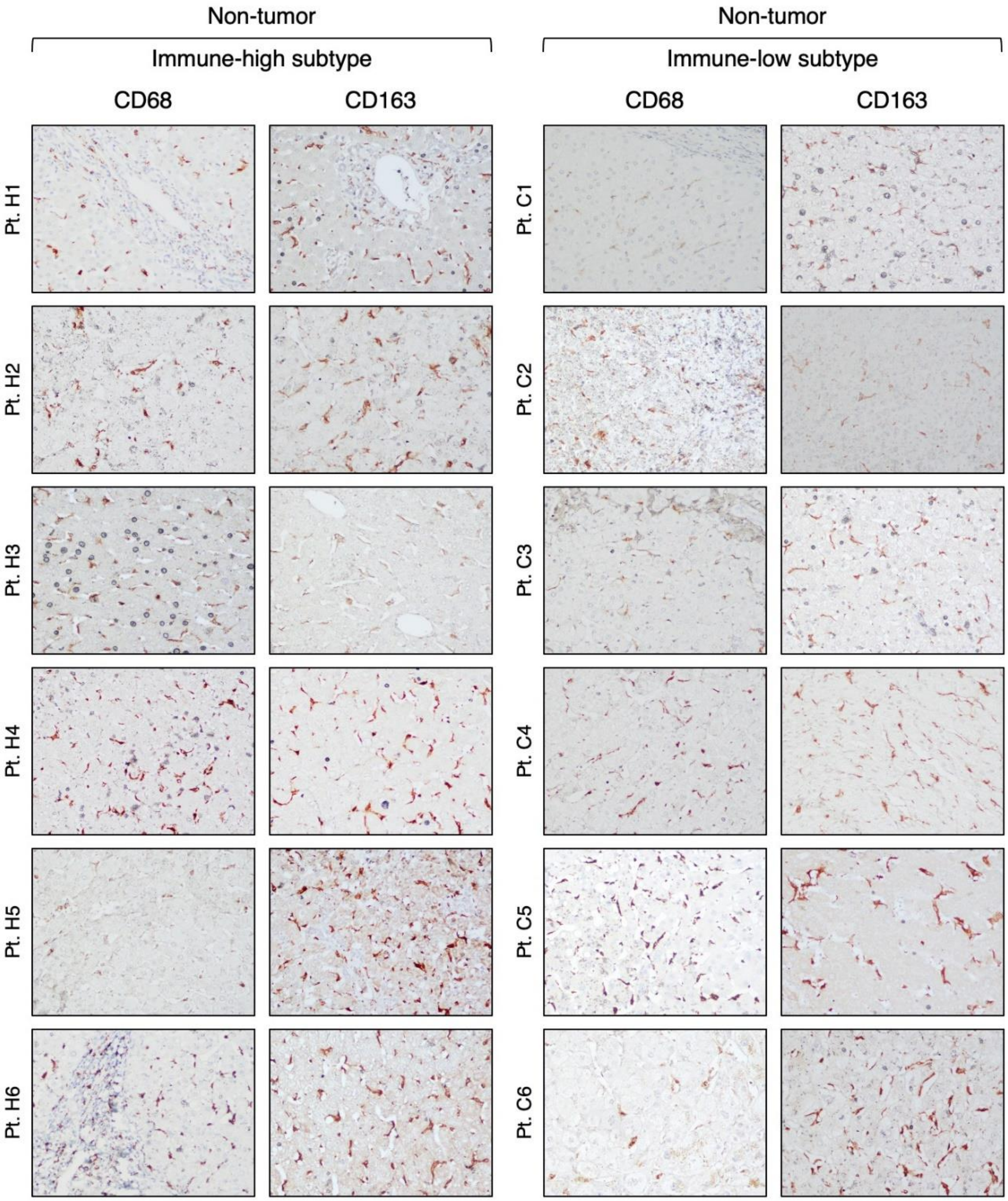

Figure S5. Immunohistochemical staining of CD68 and CD163 markers in the surrounding nontumorous tissue of immune-high and immune-low subtypes. Liver specimens obtained at the time of liver transplantation from the most distant area from the center of the tumor were stained with monoclonal antibodies against CD68 (monocytes and Kupfer cells) and CD163 (M2-like macrophages). This figure shows all patients with the immune-high subtype (left two columns) and the immune-low subtype (right two columns) in one representative field of the nontumorous tissue (200x magnification).

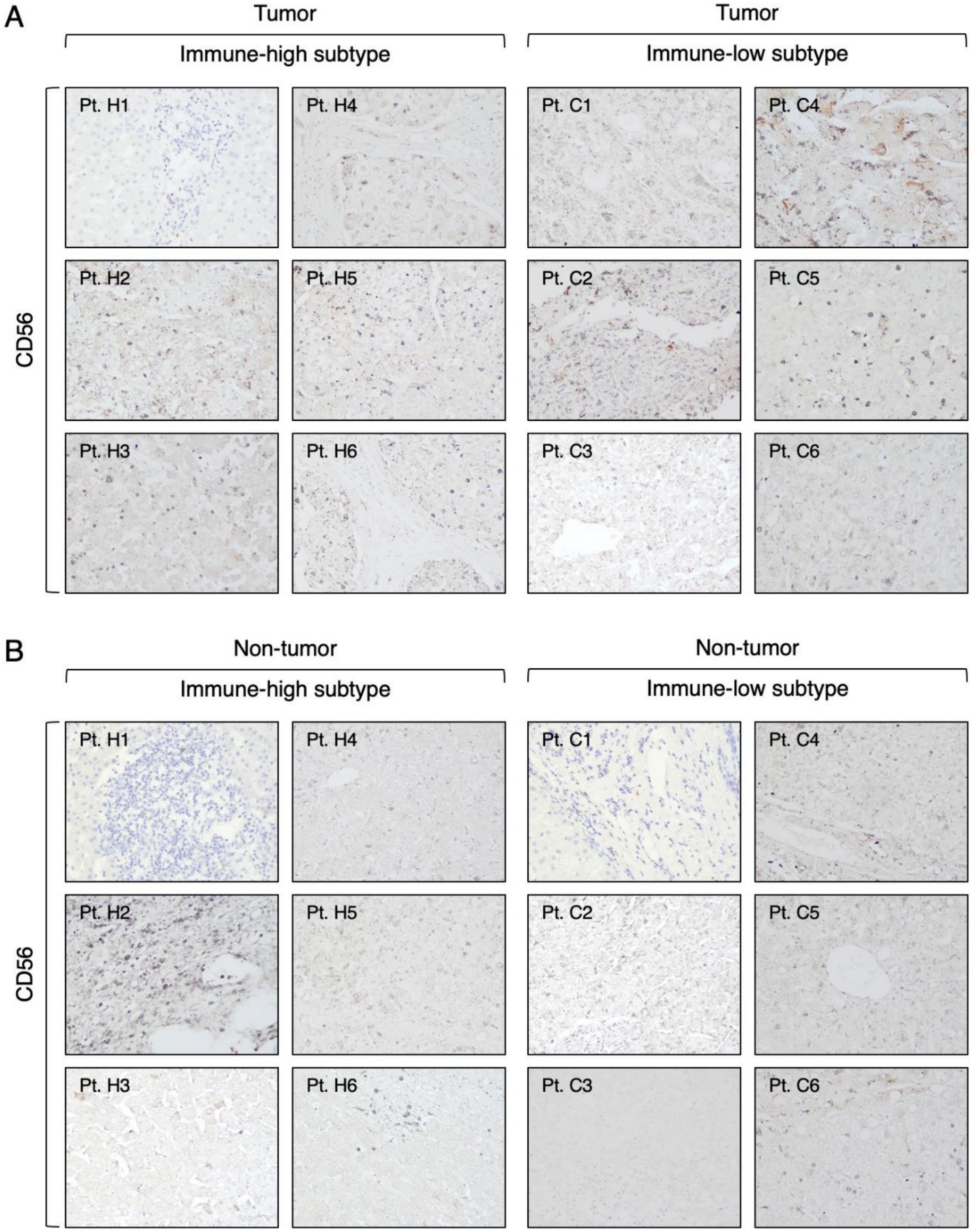

Figure S6. Expression of CD56 in the tumor and surrounding nontumorous tissue of immune-high and immune-low HCC subtypes. The images illustrate the immunostaining with monoclonal antibody against CD56 in paraffin liver sections taken at the time of liver transplantation from the center (A) and the surrounding nontumorous tissue (B). The left two columns show the immune-high subtype, and the right two columns show the immune-low subtype (200x magnification).

A

	Tumor				Non-tumor			
	Immune-high		Immune-low		Immune-high		Immune-low	
	Area A				Area C			
	H1	12	C1	2	H1	N/A	C1	N/A
	H2	12	C2	0	H2	12	C2	N/A
	H3	12	C3	8	H3	11	C3	12
	H4	10	C4	4	H4	12	C4	2
	H5	7	C5	0	H5	9	C5	12
	H6	11	C6	6	H6	9	C6	8
	Area B				Area D			
	H1	N/A	C1	N/A	H1	12	C1	4
	H2	12	C2	N/A	H2	12	C2	8
	H3	8	C3	8	H3	10	C3	7
	H4	9	C4	0	H4	4	C4	7
	H5	12	C5	4	H5	2	C5	12
	H6	8	C6	4	H6	8	C6	4
Mean ± SEM		10.3 ± 0.6		3.6 ± 0.9		9.0 ± 1.2		7.4 ± 1.3

B

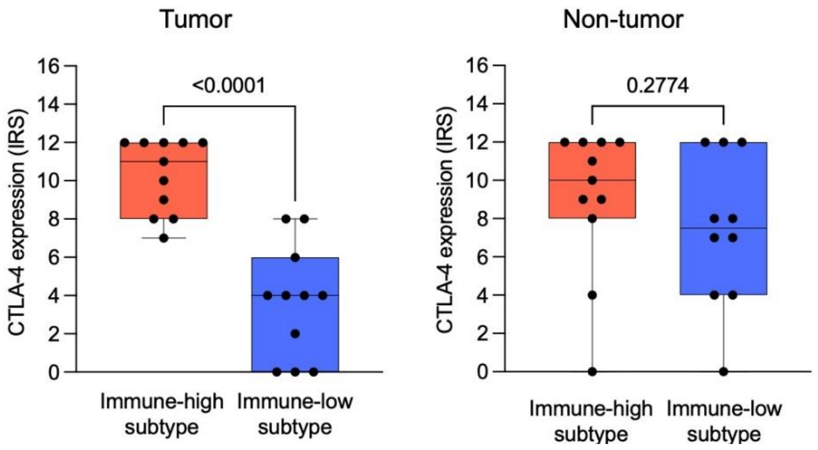

Figure S7. CTLA-4 expression in HCC and in the surrounding nontumorous tissue by IHC. (A) CTLA-4 expression by IHC in 12 HCC patients, 6 with immune-high and 6 with immune-low subtypes. For each patient, we stained two liver specimens from the tumor, one representing the center (area A) and one the periphery of the tumor (area B), and two from the nontumorous tissue, one from the perilesional area (area C) and one from the most distant area from the center of the tumor (area D, edge of the liver). The final immunoreactive score (IRS) was obtained by multiplying both percentage and intensity scores, and the values were as follow: 0-1 (negative), 2-3 (mild), 4-8 (moderate), 9-12 (strongly positive). (B) Whisker plots show the IRS of CTLA4 from each patient in different liver compartments. The median value of each subtype is indicated by a horizontal line in each box, with the 25th and 75th percentiles indicated at the top and bottom of each box; the min and max values are indicated at the top and bottom of each I bar. Statistical significance was determined using the Mann-Whitney test for the comparison between the two subtypes in the tumor (left panel) and the surrounding nontumorous tissue (right panel). N/A, denotes not available.

A

	Tumor				Non-tumor			
	Immune-high		Immune-low		Immune-high		Immune-low	
	Area A				Area C			
	H1	1	C1	0	H1	N/A	C1	N/A
	H2	1	C2	0	H2	0	C2	N/A
	H3	2	C3	0	H3	2	C3	0
	H4	2	C4	0	H4	2	C4	0
	H5	1	C5	1	H5	0	C5	1
	H6	1	C6	0	H6	1	C6	0
	Area B				Area D			
	H1	N/A	C1	N/A	H1	0	C1	1
	H2	1	C2	N/A	H2	0	C2	1
	H3	0	C3	1	H3	1	C3	0
	H4	2	C4	0	H4	1	C4	0
	H5	1	C5	0	H5	0	C5	2
	H6	0	C6	0	H6	0	C6	0
Mean ± SEM		1.4 ± 0.2		0.2 ± 0.1		0.7 ± 0.2		0.5 ± 0.2

B

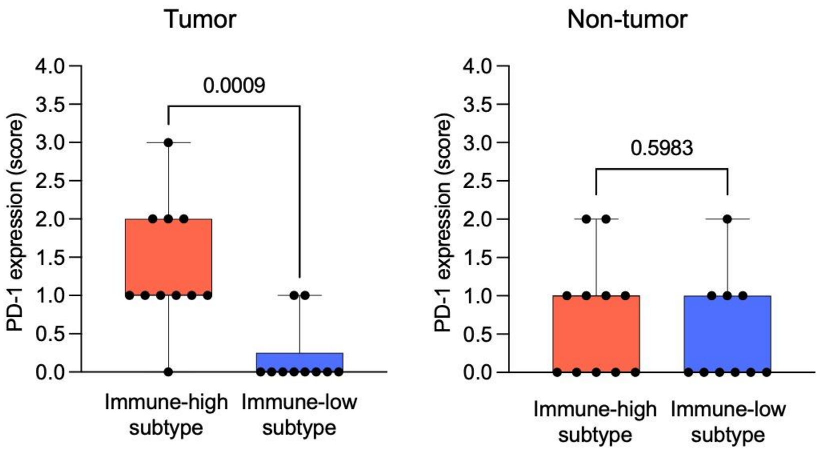

Figure S8. PD-1 expression in HCC and the surrounding nontumorous tissue analyzed by IHC. (A) PD-1 expression in 12 HCC patients, 6 with immune-high and 6 with immune-low subtypes. For each patient, we stained two liver specimens from the tumor, one representing the center (area A) and one the periphery of the tumor (area B), and two from the nontumorous tissue, one from the perilesional area (area C) and one from the most distant area from the center of the tumor (area D, edge of the liver). Semiquantitative analysis of PD-1 staining was based on the number of PD-1 positive lymphocytes: 0 (0%), 1 (<1%), 2 (1-10%), 3 (11-50%), 4 (51-90%), 5 (>90%). (B) Whisker plots show the PD-1 expression score from each patient. The median value of each subtype is indicated by a horizontal line in each box, with the 25th and 75th percentiles indicated at the top and bottom of each box; the min and max values are indicated at the top and bottom of each I bar. Statistical significance was determined using the Mann-Whitney test for the comparison between the two subtypes in the tumor (left panel) and the surrounding nontumorous tissue (right panel). N/A, denotes not available.

Table S1. List of accession numbers for RNA-seq data from individual patients with immune-high or immune-low HCC subtype.

Patient ID	Accession number	
	Tumor	Non-tumor
Immune-high subtype		
Pt. H1	SAMN18594583	SAMN18594584
Pt. H2	N/A	N/A
Pt. H3	SAMN18594593	SAMN18594594
Pt. H4	SAMN18594595	SAMN18594596
Pt. H5	SAMN18594597	SAMN18594598
Pt. H6	SAMN18594603	SAMN18594604
Immune-low subtype		
Pt. C1	SAMN18594585	SAMN18594586
Pt. C2	SAMN18594587	SAMN18594588
Pt. C3	N/A	N/A
Pt. C4	SAMN18594591	SAMN18594592
Pt. C5	SAMN18594601	SAMN18594602
Pt. C6	SAMN18594599	SAMN18594600

N/A, denotes not available.

Table S2. PD-L1 expression in HCC and the surrounding nontumorous tissue analyzed by immunohistochemistry (IHC).

Patient ID	Tumor		Non-tumor	
	Area A	Area B	Area C	Area D
Immune-high subtype				
Pt. H1	Negative	N/A	N/A	Negative
Pt. H2	Positive	Positive	Positive	Positive
Pt. H3	Positive	Negative	Positive	Positive
Pt. H4	Positive	Positive	Positive	Negative
Pt. H5	Negative	Positive	Negative	Negative
Pt. H6	Negative	Negative	Negative	Negative
Immune-low subtype				
Pt. C1	Negative	N/A	N/A	Negative
Pt. C2	Negative	N/A	N/A	Positive
Pt. C3	Positive	Positive	Positive	Positive
Pt. C4	Positive	Positive	Negative	Negative
Pt. C5	Negative	Negative	Positive	Negative
Pt. C6	Negative	Negative	Negative	Negative

PD-L1 expression by IHC in 12 HCC patients, 6 with immune-high and 6 with immune-low subtypes. For each patient, we stained two liver specimens from the tumor, one representing the center (area A) and one the periphery of the tumor (area B), and two from the nontumorous tissue, one from the perilesional area (area C) and one from the most distant area from the center of the tumor (area D, edge of the liver). PD-L1 expression was evaluated only in malignant or normal hepatocytes and the samples were considered positive if more than 1% of cells were PD-L1 positive. N/A, denotes not available.

Table S3. Genes differentially expressed in HBV-HCC patients with immune-high subtype.

Gene Symbol	Entrez Gene Name	Fold Change
MAGEA1	MAGE family member A1	9.287
RP11_463I201		8.772
ISX	intestine specific homeobox	8.588
AKR1B10	aldo-keto reductase family 1 member B10	8.202
AKR1B15	aldo-keto reductase family 1 member B15	8.136
NMRAL2P	NmrA like redox sensor 2, pseudogene	7.940
AKR1B10P1	aldo-keto reductase family 1 member B10 pseudogene 1	7.686
SLC22A12	solute carrier family 22 member 12	7.221
EPS8L3	EPS8 like 3	6.459
FSTL4	follistatin like 4	6.391
ASNSP1	asparagine synthetase pseudogene 1	6.099
TGM3	transglutaminase 3	6.099
HOTTIP	HOXA distal transcript antisense RNA	5.973
FAM133A	family with sequence similarity 133 member A	5.959
ST8SIA6-AS1	ST8SIA6 antisense RNA 1	5.957
NEIL3	nei like DNA glycosylase 3	5.834
BAGE2	BAGE family member 2	5.756
NPSR1-AS1	NPSR1 antisense RNA 1	5.720
LOC100129138	THAP domain containing 3 pseudogene	5.681
LINC02475	long intergenic non-protein coding RNA 2475	5.639
LINC01446	long intergenic non-protein coding RNA 1446	5.618
AL0784715		5.561
LINC02241		5.450
LINC02476	long intergenic non-protein coding RNA 2476	5.325
GABRD	gamma-aminobutyric acid type A receptor subunit delta	5.268
ZIC5	Zic family member 5	5.264
SPP1	secreted phosphoprotein 1	5.238
LOC730338	uncharacterized LOC730338	5.183
HTR1D	5-hydroxytryptamine receptor 1D	5.181
ZFPM2-AS1	ZFPM2 antisense RNA 1	5.150
CH17_258A224		5.098
EBF2	EBF transcription factor 2	5.072
NRCAM	neuronal cell adhesion molecule	5.031
UGT1A9 (includes others)	UDP glucuronosyltransferase family 1 member A7	5.025
CDC20	cell division cycle 20	4.959
SULT1C2	sulfotransferase family 1C member 2	4.942
TPTE	transmembrane phosphatase with tensin homology	4.827
SLC7A11	solute carrier family 7 member 11	4.772
GCNT3	glucosaminyl (N-acetyl) transferase 3, mucin type	4.751
SKA1	spindle and kinetochore associated complex subunit 1	4.743
STRA6	signaling receptor and transporter of retinol STRA6	4.730
NXPH4	neurexophilin 4	4.706
SMPX	small muscle protein X-linked	4.598
LINC02561	long intergenic non-protein coding RNA 2561	4.589
MMP11	matrix metallopeptidase 11	4.585
RP11_71H177		4.528
NEK2	NIMA related kinase 2	4.491
DEPDC1	DEP domain containing 1	4.480
TEX41	testis expressed 41	4.436
FBXW10	F-box and WD repeat domain containing 10	4.426

GSDMC	gasdermin C	4.394
UBE2C	ubiquitin conjugating enzyme E2 C	4.365
TOMM40P1	TOMM40 pseudogene 1	4.319
TTK	TTK protein kinase	4.314
FIRRE	firre intergenic repeating RNA element	4.292
CENPF	centromere protein F	4.271
SFN	stratifin	4.264
CDRT1	CMT1A duplicated region transcript 1	4.257
MKRN3	makorin ring finger protein 3	4.242
DLGAP5	DLG associated protein 5	4.214
TRIM16L	tripartite motif containing 16 like	4.203
KIF18B	kinesin family member 18B	4.184
SPC24	SPC24 component of NDC80 kinetochore complex	4.098
BUB1B	BUB1 mitotic checkpoint serine/threonine kinase B	4.061
COL24A1	collagen type XXIV alpha 1 chain	4.051
MAPK8IP2	mitogen-activated protein kinase 8 interacting protein 2	4.032
HJURP	Holliday junction recognition protein	4.016
KIF20A	kinesin family member 20A	4.011
TRIM16	tripartite motif containing 16	4.010
NUF2	NUF2 component of NDC80 kinetochore complex	3.961
CEP55	centrosomal protein 55	3.953
CRNDE	colorectal neoplasia differentially expressed	3.945
EXO1	exonuclease 1	3.943
TROAP	trophinin associated protein	3.938
RP11_146E134		3.894
NDC80	NDC80 kinetochore complex component	3.891
ACAN	aggrecan	3.811
KIF4A	kinesin family member 4A	3.801
MAFG-DT	MAFG divergent transcript	3.791
ANLN	anillin actin binding protein	3.772
OR51E1	olfactory receptor family 51 subfamily E member 1	3.769
KIF2C	kinesin family member 2C	3.764
TOP2A	DNA topoisomerase II alpha	3.707
FAM72C/FAM72D	family with sequence similarity 72 member D	3.703
SKA3	spindle and kinetochore associated complex subunit 3	3.697
KIFC1	kinesin family member C1	3.695
KIF18A	kinesin family member 18A	3.681
CDCA5	cell division cycle associated 5	3.675
CDCA8	cell division cycle associated 8	3.645
NCAPG	non-SMC condensin I complex subunit G	3.638
MAPT	microtubule associated protein tau	3.623
KIF15	kinesin family member 15	3.618
CENPM	centromere protein M	3.611
E2F8	E2F transcription factor 8	3.609
CLCNKA	chloride voltage-gated channel Ka	3.608
CDK1	cyclin dependent kinase 1	3.576
PBK	PDZ binding kinase	3.576
DUXAP9	double homeobox A pseudogene 9	3.575
DUXAP10	double homeobox A pseudogene 10	3.574
BUB1	BUB1 mitotic checkpoint serine/threonine kinase	3.563
TOB2P1	transducer of ERBB2, 2 pseudogene 1	3.553
DUXAP8	double homeobox A pseudogene 8	3.533
E2F7	E2F transcription factor 7	3.523

PTPRR	protein tyrosine phosphatase receptor type R	3.493
FOXM1	forkhead box M1	3.485
MKI67	marker of proliferation Ki-67	3.484
MELK	maternal embryonic leucine zipper kinase	3.460
BIRC5	baculoviral IAP repeat containing 5	3.456
LL22NC03-N14H11.1		3.446
CDKN3	cyclin dependent kinase inhibitor 3	3.434
HMMR	hyaluronan mediated motility receptor	3.433
GTSE1	G2 and S-phase expressed 1	3.405
TREM2	triggering receptor expressed on myeloid cells 2	3.403
KIF23	kinesin family member 23	3.391
IRX5	iroquois homeobox 5	3.372
MDK	midkine	3.340
CORIN	corin, serine peptidase	3.337
CENPA	centromere protein A	3.336
RBM24	RNA binding motif protein 24	3.316
H2AC18/H2AC19	H2A clustered histone 18	3.311
ASPM	assembly factor for spindle microtubules	3.285
KCNQ3	potassium voltage-gated channel subfamily Q member 3	3.267
SPC25	SPC25 component of NDC80 kinetochore complex	3.265
TEDC2	tubulin epsilon and delta complex 2	3.255
PTTG1	PTTG1 regulator of sister chromatid separation, securin	3.253
RP11_196G1823		3.236
CDC25C	cell division cycle 25C	3.230
POLQ	DNA polymerase theta	3.230
RP11_1055B81		3.229
HAGLR	HOXD antisense growth-associated long non-coding RNA	3.221
OLFML2B	olfactomedin like 2B	3.211
SHOX2	short stature homeobox 2	3.210
DIAPH3	diaphanous related formin 3	3.209
RHPN1-AS1	RHPN1 antisense RNA 1 (head to head)	3.207
CCNB1	cyclin B1	3.206
CAP2	cyclase associated actin cytoskeleton regulatory protein 2	3.201
EXTL1	exostosin like glycosyltransferase 1	3.183
RAB3B	RAB3B, member RAS oncogene family	3.143
RNFT2	ring finger protein, transmembrane 2	3.142
UBE2T	ubiquitin conjugating enzyme E2 T	3.138
FAM72B	family with sequence similarity 72 member B	3.135
AURKB	aurora kinase B	3.133
CCNB2	cyclin B2	3.127
TMEM74	transmembrane protein 74	3.112
TRAIP	TRAF interacting protein	3.105
EDIL3	EGF like repeats and discoidin domains 3	3.102
CCNA2	cyclin A2	3.081
FERMT1	FERM domain containing kindlin 1	3.081
CKAP2L	cytoskeleton associated protein 2 like	3.066
LPL	lipoprotein lipase	3.062
SNCG	synuclein gamma	3.032
RP11_629O12		3.029
CENPE	centromere protein E	3.002
TOMM40P2	TOMM40 pseudogene 2	3.001
SERTAD4	SERTA domain containing 4	2.993

CD109	CD109 molecule	2.985
NDUFA4L2	NDUFA4 mitochondrial complex associated like 2	2.979
TRIP13	thyroid hormone receptor interactor 13	2.966
PXDNL	peroxidasin like	2.965
PRC1	protein regulator of cytokinesis 1	2.961
CDCA3	cell division cycle associated 3	2.955
CDKN2A	cyclin dependent kinase inhibitor 2A	2.949
SPATC1L	spermatogenesis and centriole associated 1 like	2.945
PCLAF	PCNA clamp associated factor	2.939
GPX2	glutathione peroxidase 2	2.937
HRCT1	histidine rich carboxyl terminus 1	2.935
C21orf58	chromosome 21 open reading frame 58	2.929
GBAP1	glucosylceramidase beta pseudogene 1	2.923
SHCBP1	SHC binding and spindle associated 1	2.903
EME1	essential meiotic structure-specific endonuclease 1	2.881
RPLP0P2	ribosomal protein lateral stalk subunit P0 pseudogene 2	2.877
FAM83D	family with sequence similarity 83 member D	2.876
TRIM45	tripartite motif containing 45	2.863
TICRR	TOPBP1 interacting checkpoint and replication regulator	2.856
FGF13	fibroblast growth factor 13	2.852
MTFR2	mitochondrial fission regulator 2	2.824
VASH2	vasohibin 2	2.812
TDGF1	teratocarcinoma-derived growth factor 1	2.805
RP11_284F217		2.803
GAD1	glutamate decarboxylase 1	2.795
FBXO43	F-box protein 43	2.783
CENPW	centromere protein W	2.772
DUOX1	dual oxidase 1	2.769
ADM2	adrenomedullin 2	2.746
ROBO1	roundabout guidance receptor 1	2.745
IGSF3	immunoglobulin superfamily member 3	2.728
AKR1C3	aldo-keto reductase family 1 member C3	2.721
GIHCG	GIHCG inhibitor of miR-200b/200a/429 expression	2.719
KIF14	kinesin family member 14	2.714
SLC6A9	solute carrier family 6 member 9	2.707
RP11_520H147		2.704
FAM78B	family with sequence similarity 78 member B	2.701
IQGAP3	IQ motif containing GTPase activating protein 3	2.698
GINS1	GINS complex subunit 1	2.694
RP11_428P162		2.688
NOX4	NADPH oxidase 4	2.686
CAVIN4	caveolae associated protein 4	2.679
DNAJC6	DnaJ heat shock protein family (Hsp40) member C6	2.652
FAM186A	family with sequence similarity 186 member A	2.650
TONSL	tonsoku like, DNA repair protein	2.648
COL22A1	collagen type XXII alpha 1 chain	2.623
NT5DC2	5'-nucleotidase domain containing 2	2.615
ARHGEF39	Rho guanine nucleotide exchange factor 39	2.611
CBR3	carbonyl reductase 3	2.582
LINC02487		2.581
OTUB2	OTU deubiquitinase, ubiquitin aldehyde binding 2	2.577
KCNJ4	potassium inwardly rectifying channel subfamily J member 4	2.571
ZNF232-AS1	ZNF232 antisense RNA 1	2.563

ARHGAP11A	Rho GTPase activating protein 11A	2.554
RACGAP1	Rac GTPase activating protein 1	2.554
KIF11	kinesin family member 11	2.549
TKT	transketolase	2.540
PAFAH1B3	platelet activating factor acetylhydrolase 1b catalytic subunit 3	2.537
FOXS1	forkhead box S1	2.531
CAPG	capping actin protein, gelsolin like	2.518
NUSAP1	nucleolar and spindle associated protein 1	2.514
EFNA3	ephrin A3	2.503
CRYBG2	crystallin beta-gamma domain containing 2	2.481
MND1	meiotic nuclear divisions 1	2.478
LINC01134	long intergenic non-protein coding RNA 1134	2.472
HES6	hes family bHLH transcription factor 6	2.449
UGT1A6	UDP glucuronosyltransferase family 1 member A6	2.447
FABP5	fatty acid binding protein 5	2.444
CDKN2B-AS1	CDKN2B antisense RNA 1	2.443
CENPI	centromere protein I	2.438
PIF1	PIF1 5'-to-3' DNA helicase	2.438
TMCC1-DT	TMCC1 divergent transcript	2.430
MSX1	msh homeobox 1	2.409
RECQL4	RecQ like helicase 4	2.406
SLC26A6	solute carrier family 26 member 6	2.404
TK1	thymidine kinase 1	2.397
OSBP2	oxysterol binding protein 2	2.395
MAPK12	mitogen-activated protein kinase 12	2.393
PARPBP	PARP1 binding protein	2.370
PIR	pirin	2.364
MINCR	MYC-induced long non-coding RNA	2.353
CCNF	cyclin F	2.344
UBD	ubiquitin D	2.341
CAPN11	calpain 11	2.338
RP11_793H1311		2.332
PRKAA2	protein kinase AMP-activated catalytic subunit alpha 2	2.330
ARHGEF2-AS2	ARHGEF2 antisense RNA 2	2.315
EFNA4	ephrin A4	2.312
LOC105370941	uncharacterized LOC105370941	2.307
NFE2L1-DT		2.305
MSANTD3-TMEFF1	MSANTD3-TMEFF1 readthrough	2.303
ZSWIM5	zinc finger SWIM-type containing 5	2.303
ARHGAP39	Rho GTPase activating protein 39	2.301
CHRDL2	chordin like 2	2.300
RASD2	RASD family member 2	2.297
HSPB1	heat shock protein family B (small) member 1	2.284
SPSB2	splA/ryanodine receptor domain and SOCS box containing 2	2.275
RRM2	ribonucleotide reductase regulatory subunit M2	2.265
GBA	glucosylceramidase beta	2.253
RASL12	RAS like family 12	2.253
ADAMTS14	ADAM metallopeptidase with thrombospondin type 1 motif 14	2.248
RAD54B	RAD54 homolog B	2.244
THY1	Thy-1 cell surface antigen	2.244
STMN1	stathmin 1	2.240
TP53I3	tumor protein p53 inducible protein 3	2.231
HSPB7	heat shock protein family B (small) member 7	2.229

TXNRD1	thioredoxin reductase 1	2.225
GJC1	gap junction protein gamma 1	2.222
MACIR	macrophage immunometabolism regulator	2.221
FIGNL2	fidgetin like 2	2.208
PRKAR2A-AS1	PRKAR2A antisense RNA 1	2.185
HDAC11	histone deacetylase 11	2.181
HEXA-AS1	HEXA antisense RNA 1	2.178
TTLL1	tubulin tyrosine ligase like 1	2.171
CACNG8	calcium voltage-gated channel auxiliary subunit gamma 8	2.165
TRPC6	transient receptor potential cation channel subfamily C member 6	2.158
MTBP	MDM2 binding protein	2.156
LOC107984285	uncharacterized LOC107984285	2.154
STK39	serine/threonine kinase 39	2.153
APOBEC3B	apolipoprotein B mRNA editing enzyme catalytic subunit 3B	2.148
ARHGAP22	Rho GTPase activating protein 22	2.146
TMEM108	transmembrane protein 108	2.137
DSCC1	DNA replication and sister chromatid cohesion 1	2.133
CCDC34	coiled-coil domain containing 34	2.127
MAD2L1	mitotic arrest deficient 2 like 1	2.127
AC068831.7		2.120
RHBDL1	rhomboid like 1	2.120
ANKRD13B	ankyrin repeat domain 13B	2.118
H4C15	H4 clustered histone 15	2.112
H2BC19P	H2B clustered histone 19, pseudogene	2.106
LINC01572	long intergenic non-protein coding RNA 1572	2.106
TCF19	transcription factor 19	2.106
OLFML2A	olfactomedin like 2A	2.101
RP11-704M14.1		2.100
TMEM106C	transmembrane protein 106C	2.099
RIMBP3 (includes others)	RIMS binding protein 3	2.084
ZBTB12	zinc finger and BTB domain containing 12	2.070
TMEM74B	transmembrane protein 74B	2.067
NOTCH3	notch receptor 3	2.059
DLG5	discs large MAGUK scaffold protein 5	2.053
EHMT2	euchromatic histone lysine methyltransferase 2	2.040
PANX2	pannexin 2	2.039
RP11_5O171		2.037
SPON2	spondin 2	2.033
STIL	STIL centriolar assembly protein	2.032
CDH24	cadherin 24	2.023
LINGO1	leucine rich repeat and Ig domain containing 1	2.021
RP11_651L53		2.017
RP1_140K85		2.014
HRAT92	heart tissue-associated transcript 92	2.011
DNMT3B	DNA methyltransferase 3 beta	2.010
SAC3D1	SAC3 domain containing 1	2.008
FBXO32	F-box protein 32	2.003
RP11_531A245		1.997
RNF157	ring finger protein 157	1.992
WDR62	WD repeat domain 62	1.991
STAM-DT	STAM divergent transcript	1.989
AKR1C1/AKR1C2	aldo-keto reductase family 1 member C2	1.987

SEPTIN5	septin 5	1.982
CTC_471F35		1.960
RRAGD	Ras related GTP binding D	1.960
EBF1	EBF transcription factor 1	1.958
SEPT5-GP1BB	SEPT5-GP1BB readthrough	1.957
RAC3	Rac family small GTPase 3	1.955
NUDT1	nudix hydrolase 1	1.953
DDIAS	DNA damage induced apoptosis suppressor	1.940
CAVIN3	caveolae associated protein 3	1.939
GOLGA2P10	GOLGA2 pseudogene 10	1.939
CEP131	centrosomal protein 131	1.937
CTD_3065J169		1.936
RP11_119F75		1.931
CPT1C	carnitine palmitoyltransferase 1C	1.928
ATXN7L3-AS1	ATXN7L3 antisense RNA 1	1.915
PLVAP	plasmalemma vesicle associated protein	1.902
GLMP	glycosylated lysosomal membrane protein	1.891
HOMER3	homer scaffold protein 3	1.890
FAM189B	family with sequence similarity 189 member B	1.887
C5orf34	chromosome 5 open reading frame 34	1.886
RP11-810P12.7		1.885
ZNF252P-AS1	ZNF252P antisense RNA 1	1.883
GLI4	GLI family zinc finger 4	1.882
CD34	CD34 molecule	1.880
RASA4DP	RAS p21 protein activator 4CD, pseudogene	1.880
S100A10	S100 calcium binding protein A10	1.873
DCAF4L1	DDB1 and CUL4 associated factor 4 like 1	1.872
ASB16	ankyrin repeat and SOCS box containing 16	1.869
NSMCE2	NSE2 (MMS21) homolog, SMC5-SMC6 complex SUMO ligase	1.867
BICDL1	BICD family like cargo adaptor 1	1.865
RP11_412D94		1.862
SERPINI1	serpin family I member 1	1.862
MIF	macrophage migration inhibitory factor	1.860
RP11_392O172		1.857
PVT1	Pvt1 oncogene	1.846
B3GALNT1	beta-1,3-N-acetylgalactosaminyltransferase 1 (globoside blood group)	1.845
SLC38A6	solute carrier family 38 member 6	1.842
CSPG4	chondroitin sulfate proteoglycan 4	1.838
NRGN	neurogranin	1.836
RP11_16N112		1.833
NR2C2AP	nuclear receptor 2C2 associated protein	1.828
ATP6V0E2-AS1	ATP6V0E2 antisense RNA 1	1.820
ENOX1	ecto-NOX disulfide-thiol exchanger 1	1.820
SRXN1	sulfiredoxin 1	1.815
LOC101930100	uncharacterized LOC101930100	1.810
HOXB5	homeobox B5	1.808
FSD1L	fibronectin type III and SPRY domain containing 1 like	1.805
SEMA5B	semaphorin 5B	1.802
TRIM6	tripartite motif containing 6	1.800
MTHFD1L	methylenetetrahydrofolate dehydrogenase (NADP+ dependent) 1 like	1.798
PLXDC1	plexin domain containing 1	1.797
UBE2S	ubiquitin conjugating enzyme E2 S	1.797
RP11_181E103		1.795

DTNA	dystrobrevin alpha	1.790
CITF22_92A61		1.786
AC022007.1		1.784
SPA17	sperm autoantigenic protein 17	1.784
FAM21FP	family with sequence similarity 21 member F, pseudogene	1.782
SOWAHCP2	SOWAHC pseudogene 2	1.781
LOC100996419	uncharacterized LOC100996419	1.774
CORO6	coronin 6	1.773
GPAA1	glycosylphosphatidylinositol anchor attachment 1	1.767
TBX6	T-box transcription factor 6	1.757
ANKRD20A17P	ankyrin repeat domain 20 family member A17, pseudogene	1.756
RP3_412A916		1.755
FLJ46284	uncharacterized LOC441369	1.754
TNFRSF4	TNF receptor superfamily member 4	1.753
RNF187	ring finger protein 187	1.747
RTL8B	retrotransposon Gag like 8B	1.741
ASAP3	ArfGAP with SH3 domain, ankyrin repeat and PH domain 3	1.739
CENPU	centromere protein U	1.731
RP11_158H58		1.730
ADAMTS7	ADAM metallopeptidase with thrombospondin type 1 motif 7	1.725
PRXL2B	peroxiredoxin like 2B	1.721
CTSA	cathepsin A	1.720
SCRIB	scribble planar cell polarity protein	1.717
ASPH	aspartate beta-hydroxylase	1.715
BOLA2/BOLA2B	bolA family member 2	1.713
PLCD4	phospholipase C delta 4	1.710
MAP2	microtubule associated protein 2	1.704
PIK3R2	phosphoinositide-3-kinase regulatory subunit 2	1.702
RFXANK	regulatory factor X associated ankyrin containing protein	1.702
BCL9	BCL9 transcription coactivator	1.700
PABPC4L	poly(A) binding protein cytoplasmic 4 like	1.696
RGS5	regulator of G protein signaling 5	1.693
NCAPD2	non-SMC condensin I complex subunit D2	1.691
ANO10	anoctamin 10	1.683
SPDL1	spindle apparatus coiled-coil protein 1	1.683
ZBED8	zinc finger BED-type containing 8	1.682
LINC02767	long intergenic non-protein coding RNA 2767	1.677
N4BP3	NEDD4 binding protein 3	1.677
PYGB	glycogen phosphorylase B	1.677
LLGL1	LLGL scribble cell polarity complex component 1	1.671
HTATIP2	HIV-1 Tat interactive protein 2	1.664
CENPX	centromere protein X	1.657
DPY19L1P1	DPY19L1 pseudogene 1	1.657
CENPQ	centromere protein Q	1.654
RNASEH2A	ribonuclease H2 subunit A	1.651
CCDC24	coiled-coil domain containing 24	1.650
RHNO1	RAD9-HUS1-RAD1 interacting nuclear orphan 1	1.642
GRIN2D	glutamate ionotropic receptor NMDA type subunit 2D	1.639
KMT5C	lysine methyltransferase 5C	1.631
AGTRAP	angiotensin II receptor associated protein	1.629
DUT	deoxyuridine triphosphatase	1.628
DNM3	dynamin 3	1.626
PTP4A3	protein tyrosine phosphatase 4A3	1.624

CTA_445C914		1.622
H2BC20P	H2B clustered histone 20, pseudogene	1.621
ZNF775	zinc finger protein 775	1.617
CD2BP2-DT	CD2BP2 divergent transcript	1.616
LRRC45	leucine rich repeat containing 45	1.616
AC0093032		1.612
ZNF572	zinc finger protein 572	1.612
BOP1	BOP1 ribosomal biogenesis factor	1.611
MFSD3	major facilitator superfamily domain containing 3	1.611
KRT8P12	keratin 8 pseudogene 12	1.605
JAG2	jagged canonical Notch ligand 2	1.601
TPM2	tropomyosin 2	1.597
GOLM1	golgi membrane protein 1	1.594
RP3_406P245		1.594
ITGA7	integrin subunit alpha 7	1.592
KIFC2	kinesin family member C2	1.586
CDPF1P1	CDPF1 pseudogene 1	1.581
CCDC77	coiled-coil domain containing 77	1.580
RP11_1017G215		1.580
TARBP1	TAR (HIV-1) RNA binding protein 1	1.579
HGH1	HGH1 homolog	1.574
ZC2HC1A	zinc finger C2HC-type containing 1A	1.571
HSF4	heat shock transcription factor 4	1.570
RP11_18H71		1.568
MSH5-SAPCD1	MSH5-SAPCD1 readthrough (NMD candidate)	1.565
ERICH2	glutamate rich 2	1.557
LAGE3	L antigen family member 3	1.550
PDE1C	phosphodiesterase 1C	1.549
COA6	cytochrome c oxidase assembly factor 6	1.543
LOC283710		1.543
CDK13-DT	CDK13 divergent transcript	1.542
FBF1	Fas binding factor 1	1.542
TBC1D31	TBC1 domain family member 31	1.541
PLEKHA8P1	pleckstrin homology domain containing A8 pseudogene 1	1.540
FOXRED2	FAD dependent oxidoreductase domain containing 2	1.539
SCARA3	scavenger receptor class A member 3	1.538
RP11_600F247		1.536
ZFP41	ZFP41 zinc finger protein	1.536
OXLD1	oxidoreductase like domain containing 1	1.535
CLCN7	chloride voltage-gated channel 7	1.532
ENAH	ENAH actin regulator	1.529
CCHCR1	coiled-coil alpha-helical rod protein 1	1.528
AMDHD2	amidohydrolase domain containing 2	1.521
RP11_546D63		1.520
TUBA1B	tubulin alpha 1b	1.520
FAM110A	family with sequence similarity 110 member A	1.515
CMB9-55F22.1		1.514
CHN1	chimerin 1	1.512
H2AC6	H2A clustered histone 6	1.511
TLCD1	TLC domain containing 1	1.511
LINC02381	long intergenic non-protein coding RNA 2381	1.505
PRIM2	DNA primase subunit 2	1.505
LOC100287896		1.502

TLCD5	TLC domain containing 5	1.502
C7orf25	chromosome 7 open reading frame 25	1.500
LINC01011	long intergenic non-protein coding RNA 1011	1.500
C20orf27	chromosome 20 open reading frame 27	1.499
C18orf54	chromosome 18 open reading frame 54	1.498
SLC52A2	solute carrier family 52 member 2	1.497
RP11_197N182		1.495
MFGE8	milk fat globule EGF and factor V/VIII domain containing	1.492
SFR1	SWI5 dependent homologous recombination repair protein 1	1.490
BBLN	bublin coiled coil protein	1.483
LOC100507437	uncharacterized LOC100507437	1.480
ABCC5	ATP binding cassette subfamily C member 5	1.479
BOLA1	bolA family member 1	1.475
C20orf96	chromosome 20 open reading frame 96	1.475
ZKSCAN3	zinc finger with KRAB and SCAN domains 3	1.473
ANXA2P2	annexin A2 pseudogene 2	1.467
FRS3	fibroblast growth factor receptor substrate 3	1.465
ROBO3	roundabout guidance receptor 3	1.465
TAF6	TATA-box binding protein associated factor 6	1.465
BAG2	BAG cochaperone 2	1.463
PYCR3	pyrroline-5-carboxylate reductase 3	1.463
ARL2	ADP ribosylation factor like GTPase 2	1.462
SNRPE	small nuclear ribonucleoprotein polypeptide E	1.460
THAP8	THAP domain containing 8	1.457
FAAP24	FA core complex associated protein 24	1.455
DSTNP2	DSTN pseudogene 2	1.454
KDM4D	lysine demethylase 4D	1.453
DVL2	dishevelled segment polarity protein 2	1.444
CTA_228A94		1.440
EXOSC4	exosome component 4	1.438
SCNM1	sodium channel modifier 1	1.437
PACC1	proton activated chloride channel 1	1.436
IQCC	IQ motif containing C	1.433
LOC171391	uncharacterized LOC171391	1.430
ZNF213	zinc finger protein 213	1.429
MSH2	mutS homolog 2	1.428
CKAP2	cytoskeleton associated protein 2	1.427
SQSTM1	sequestosome 1	1.427
NDRG3	NDRG family member 3	1.426
POLA2	DNA polymerase alpha 2, accessory subunit	1.424
RPL13P5	ribosomal protein L13 pseudogene 5	1.423
FHIT	fragile histidine triad diadenosine triphosphatase	1.422
IFT81	intraflagellar transport 81	1.421
PLA2G6	phospholipase A2 group VI	1.417
CDPF1	cysteine rich DPF motif domain containing 1	1.416
MANEAL	mannosidase endo-alpha like	1.416
ANXA2	annexin A2	1.415
HEXA	hexosaminidase subunit alpha	1.414
MSTO1	misato mitochondrial distribution and morphology regulator 1	1.414
C1orf35	chromosome 1 open reading frame 35	1.412
DBP	D-box binding PAR bZIP transcription factor	1.411
FTL	ferritin light chain	1.410
NUP37	nucleoporin 37	1.408

ZBTB22	zinc finger and BTB domain containing 22	1.408
EID2B	EP300 interacting inhibitor of differentiation 2B	1.407
TMEM81	transmembrane protein 81	1.407
ANTKMT	adenine nucleotide translocase lysine methyltransferase	1.405
SMIM29	small integral membrane protein 29	1.403
PODXL	podocalyxin like	1.402
RP5_1074L14		1.401
BBC3	BCL2 binding component 3	1.398
SAMD10	sterile alpha motif domain containing 10	1.391
ARHGAP33	Rho GTPase activating protein 33	1.386
CSTB	cystatin B	1.386
NME1	NME/NM23 nucleoside diphosphate kinase 1	1.385
TALDO1	transaldolase 1	1.385
AZIN2	antizyme inhibitor 2	1.384
CDK4	cyclin dependent kinase 4	1.384
KNSTRN	kinetochore localized astrin (SPAG5) binding protein	1.384
SPATS2	spermatogenesis associated serine rich 2	1.384
P4HA2	prolyl 4-hydroxylase subunit alpha 2	1.383
CDK5	cyclin dependent kinase 5	1.382
ATRIP	ATR interacting protein	1.381
TUBG1	tubulin gamma 1	1.380
ACBD6	acyl-CoA binding domain containing 6	1.379
MSTO2P	misato family member 2, pseudogene	1.378
PFDN6	prefoldin subunit 6	1.378
CUEDC1	CUE domain containing 1	1.377
KCTD1	potassium channel tetramerization domain containing 1	1.376
AGRN	agrin	1.375
SHARPIN	SHANK associated RH domain interactor	1.371
ABHD4	abhydrolase domain containing 4, N-acyl phospholipase B	1.370
DTYMK	deoxythymidylate kinase	1.370
PGF	placental growth factor	1.369
CERS6-AS1	CERS6 antisense RNA 1	1.368
NECAB3	N-terminal EF-hand calcium binding protein 3	1.366
SF3B4	splicing factor 3b subunit 4	1.366
TSPAN15	tetraspanin 15	1.363
VPS72	vacuolar protein sorting 72 homolog	1.362
MIS18A	MIS18 kinetochore protein A	1.359
SHLD1	shieldin complex subunit 1	1.359
XXYLT1	xyloside xylosyltransferase 1	1.359
TXN	thioredoxin	1.358
PCSK5	proprotein convertase subtilisin/kexin type 5	1.354
LOXL2	lysyl oxidase like 2	1.349
KIAA1614	KIAA1614	1.348
TMEM201	transmembrane protein 201	1.348
ZSCAN2	zinc finger and SCAN domain containing 2	1.348
PROCA1	protein interacting with cyclin A1	1.347
PSMD4	proteasome 26S subunit, non-ATPase 4	1.347
RP11_513I156		1.345
PPP1R16A	protein phosphatase 1 regulatory subunit 16A	1.342
ZNF43	zinc finger protein 43	1.342
ADAM15	ADAM metallopeptidase domain 15	1.339
SDF2L1	stromal cell derived factor 2 like 1	1.339
SPACA9	sperm acrosome associated 9	1.338

NUDT14	nudix hydrolase 14	1.337
ITGA6	integrin subunit alpha 6	1.334
SMYD3	SET and MYND domain containing 3	1.334
STPG1	sperm tail PG-rich repeat containing 1	1.334
NABP2	nucleic acid binding protein 2	1.332
ZNF517	zinc finger protein 517	1.329
LINC00910	long intergenic non-protein coding RNA 910	1.328
H2AZ1	H2A.Z variant histone 1	1.326
TEAD2	TEA domain transcription factor 2	1.326
MIF4GD-DT	MIF4GD divergent transcript	1.325
FBXL19	F-box and leucine rich repeat protein 19	1.324
ATP6V1H	ATPase H+ transporting V1 subunit H	1.322
GLB1L	galactosidase beta 1 like	1.322
ALS2CL	ALS2 C-terminal like	1.321
FAM83H	family with sequence similarity 83 member H	1.320
SCAMP3	secretory carrier membrane protein 3	1.320
PIGC	phosphatidylinositol glycan anchor biosynthesis class C	1.319
ADA	adenosine deaminase	1.314
B3GNTL1	UDP-GlcNAc:betaGal beta-1,3-N-acetylglucosaminyltransferase like 1	1.312
CSPG4P10	chondroitin sulfate proteoglycan 4 pseudogene 10	1.312
PPOX	protoporphyrinogen oxidase	1.312
ZNF692	zinc finger protein 692	1.312
CD63-AS1	CD63 antisense RNA 1	1.307
CTB_52I24		1.307
LINC01772	long intergenic non-protein coding RNA 1772	1.307
ZEB1-AS1	ZEB1 antisense RNA 1	1.306
COMMD4	COMM domain containing 4	1.305
HCN3	hyperpolarization activated cyclic nucleotide gated potassium channel 3	1.304
ADCK5	aarF domain containing kinase 5	1.303
AKIP1	A-kinase interacting protein 1	1.302
CYHR1	cysteine and histidine rich 1	1.302
KDM4A-AS1	KDM4A antisense RNA 1	1.301
NAGPA	N-acetylglucosamine-1-phosphodiester alpha-N-acetylglucosaminidase	1.294
BICD1	BICD cargo adaptor 1	1.293
CTD_2006H142		1.293
RPUSD1	RNA pseudouridine synthase domain containing 1	1.291
CPLANE2	ciliogenesis and planar polarity effector 2	1.289
GFUS	GDP-L-fucose synthase	1.288
SRP14-AS1	SRP14 antisense RNA1 (head to head)	1.285
DPM3	dolichyl-phosphate mannosyltransferase subunit 3, regulatory	1.278
LINC01126	long intergenic non-protein coding RNA 1126	1.275
PLEKHH3	pleckstrin homology, MyTH4 and FERM domain containing H3	1.275
LINC01089	long intergenic non-protein coding RNA 1089	1.273
PPP1R14B	protein phosphatase 1 regulatory inhibitor subunit 14B	1.273
SLC41A3	solute carrier family 41 member 3	1.272
ASB16-AS1	ASB16 antisense RNA 1	1.271
PFDN4	prefoldin subunit 4	1.271
RP5_1024N44		1.271
BAMBI	BMP and activin membrane bound inhibitor	1.265
PYGO2	pygopus family PHD finger 2	1.258
LOC105369748		1.257
SVBP	small vasohibin binding protein	1.256
FKBPL	FKBP prolyl isomerase like	1.255

CSTF2	cleavage stimulation factor subunit 2	1.253
AIFM2	apoptosis inducing factor mitochondria associated 2	1.252
APIP	APAF1 interacting protein	1.252
SAMD1	sterile alpha motif domain containing 1	1.252
UQCC2	ubiquinol-cytochrome c reductase complex assembly factor 2	1.252
AP5Z1	adaptor related protein complex 5 subunit zeta 1	1.250
FBXL22	F-box and leucine rich repeat protein 22	1.250
SLC48A1	solute carrier family 48 member 1	1.250
CYB5R1	cytochrome b5 reductase 1	1.247
DNAL4	dynein axonemal light chain 4	1.244
C3orf18	chromosome 3 open reading frame 18	1.241
ZNF174	zinc finger protein 174	1.241
NOL3	nucleolar protein 3	1.240
RP1_39G227		1.237
DALRD3	DALR anticodon binding domain containing 3	1.232
FBXO44	F-box protein 44	1.230
LOC101927151	uncharacterized LOC101927151	1.230
LRRC14	leucine rich repeat containing 14	1.230
NELFE	negative elongation factor complex member E	1.229
CCDC15	coiled-coil domain containing 15	1.225
FGD1	FYVE, RhoGEF and PH domain containing 1	1.223
OGFOD2	2-oxoglutarate and iron dependent oxygenase domain containing 2	1.223
PLOD1	procollagen-lysine,2-oxoglutarate 5-dioxygenase 1	1.223
TUBG2	tubulin gamma 2	1.223
DCLRE1B	DNA cross-link repair 1B	1.221
SPNS1	sphingolipid transporter 1 (putative)	1.219
DENND6B	DENN domain containing 6B	1.218
SNHG30	small nucleolar RNA host gene 30	1.218
HEY1	hes related family bHLH transcription factor with YRPW motif 1	1.217
RHBDD3	rhomboid domain containing 3	1.217
SNRPC	small nuclear ribonucleoprotein polypeptide C	1.216
TMEM101	transmembrane protein 101	1.215
ZNF74	zinc finger protein 74	1.214
MAPKAPK5-AS1	MAPKAPK5 antisense RNA 1	1.212
OSBPL3	oxysterol binding protein like 3	1.212
MAF1	MAF1 homolog, negative regulator of RNA polymerase III	1.211
PPP1R35	protein phosphatase 1 regulatory subunit 35	1.211
SH3BP5L	SH3 binding domain protein 5 like	1.211
NFKBIL1	NFKB inhibitor like 1	1.208
TMEM164	transmembrane protein 164	1.207
AACS	acetoacetyl-CoA synthetase	1.204
IDUA	alpha-L-iduronidase	1.202
SELENON	selenoprotein N	1.200
SNX15	sorting nexin 15	1.200
BAX	BCL2 associated X, apoptosis regulator	1.199
PIGU	phosphatidylinositol glycan anchor biosynthesis class U	1.199
C8orf33	chromosome 8 open reading frame 33	1.197
PRPF40B	pre-mRNA processing factor 40 homolog B	1.196
ZNNT1	ZNF706 neighboring transcript 1	1.196
MCAM	melanoma cell adhesion molecule	1.193
GNPDA1	glucosamine-6-phosphate deaminase 1	1.192
AC009133.12		1.191
CLN6	CLN6 transmembrane ER protein	1.191

RCCD1	RCC1 domain containing 1	1.188
RUSC1	RUN and SH3 domain containing 1	1.188
MEA1	male-enhanced antigen 1	1.187
HSP90AB1	heat shock protein 90 alpha family class B member 1	1.185
HNRNPA1P16	heterogeneous nuclear ribonucleoprotein A1 pseudogene 16	1.184
VPS45	vacuolar protein sorting 45 homolog	1.184
B4GALT6	beta-1,4-galactosyltransferase 6	1.183
MID1IP1	MID1 interacting protein 1	1.180
SLC25A39	solute carrier family 25 member 39	1.180
CEP41	centrosomal protein 41	1.178
SMIM4	small integral membrane protein 4	1.176
ATP13A2	ATPase cation transporting 13A2	1.175
CCDC163	coiled-coil domain containing 163	1.171
CEP85	centrosomal protein 85	1.170
TMEM9	transmembrane protein 9	1.168
AJM1	apical junction component 1 homolog	1.163
NTPCR	nucleoside-triphosphatase, cancer-related	1.162
SLC16A1-AS1	SLC16A1 antisense RNA 1	1.156
NVL	nuclear VCP like	1.155
FMNL3	formin like 3	1.153
MLST8	MTOR associated protein, LST8 homolog	1.143
MFSD5	major facilitator superfamily domain containing 5	1.142
CEP152	centrosomal protein 152	1.141
CCT3	chaperonin containing TCP1 subunit 3	1.139
ZFP64	ZFP64 zinc finger protein	1.134
PRKDC	protein kinase, DNA-activated, catalytic subunit	1.133
PRDX1	peroxiredoxin 1	1.131
HNRNPCP7	heterogeneous nuclear ribonucleoprotein C pseudogene 7	1.129
EVI5L	ecotropic viral integration site 5 like	1.126
MAN1B1-DT	MAN1B1 divergent transcript	1.126
VPS28	VPS28 subunit of ESCRT-I	1.126
MAPK3	mitogen-activated protein kinase 3	1.125
ATP6V1E2	ATPase H+ transporting V1 subunit E2	1.124
BCAP31	B cell receptor associated protein 31	1.123
EVA1B	eva-1 homolog B	1.123
GPR137	G protein-coupled receptor 137	1.123
C7orf50	chromosome 7 open reading frame 50	1.120
H2AJ	H2A.J histone	1.120
TMEM79	transmembrane protein 79	1.120
ARPC1B	actin related protein 2/3 complex subunit 1B	1.118
TRAF7	TNF receptor associated factor 7	1.118
CLN3	CLN3 lysosomal/endosomal transmembrane protein, battenin	1.113
SNRPA	small nuclear ribonucleoprotein polypeptide A	1.113
MAZ	MYC associated zinc finger protein	1.111
STOML1	stomatin like 1	1.110
WASF1	WASP family member 1	1.110
G6PC3	glucose-6-phosphatase catalytic subunit 3	1.109
KATNB1	katanin regulatory subunit B1	1.108
SOGA1	suppressor of glucose, autophagy associated 1	1.108
CD320	CD320 molecule	1.107
PDGFB	platelet derived growth factor subunit B	1.106
PDIA3P1	protein disulfide isomerase family A member 3 pseudogene 1	1.106
NTAQ1	N-terminal glutamine amidase 1	1.105

TBKBP1	TBK1 binding protein 1	1.105
ARSA	arylsulfatase A	1.104
WASHC5	WASH complex subunit 5	1.104
CDKN2AIPNL	CDKN2A interacting protein N-terminal like	1.102
ZNF707	zinc finger protein 707	1.102
COG2	component of oligomeric golgi complex 2	1.101
SERPINH1	serpin family H member 1	1.100
HMGN4	high mobility group nucleosomal binding domain 4	1.099
SLC50A1	solute carrier family 50 member 1	1.099
ZNF337-AS1	ZNF337 antisense RNA 1	1.098
ZNF219	zinc finger protein 219	1.097
MRGBP	MRG domain binding protein	1.095
HRAS	HRas proto-oncogene, GTPase	1.094
PLOD3	procollagen-lysine,2-oxoglutarate 5-dioxygenase 3	1.092
RUVBL1	RuvB like AAA ATPase 1	1.091
FLVCR1	FLVCR heme transporter 1	1.090
TUBA4A	tubulin alpha 4a	1.090
TRIM62	tripartite motif containing 62	1.089
MZT1	mitotic spindle organizing protein 1	1.087
ATP5MF-PTCD1	ATP5MF-PTCD1 readthrough	1.086
GTPBP2	GTP binding protein 2	1.086
ZNF691	zinc finger protein 691	1.085
DET1	DET1 partner of COP1 E3 ubiquitin ligase	1.083
HPS4	HPS4 biogenesis of lysosomal organelles complex 3 subunit 2	1.081
PIK3C2B	phosphatidylinositol-4-phosphate 3-kinase catalytic subunit type 2 beta	1.081
ATF6B	activating transcription factor 6 beta	1.080
ZNF605	zinc finger protein 605	1.080
PTGFRN	prostaglandin F2 receptor inhibitor	1.078
TSEN15	tRNA splicing endonuclease subunit 15	1.078
NME2	NME/NM23 nucleoside diphosphate kinase 2	1.077
PIAS3	protein inhibitor of activated STAT 3	1.076
NAA40	N-alpha-acetyltransferase 40, NatD catalytic subunit	1.075
CNPY4	canopy FGF signaling regulator 4	1.074
ZNF668	zinc finger protein 668	1.074
CFL1	cofilin 1	1.070
TMEM42	transmembrane protein 42	1.070
RTL8C	retrotransposon Gag like 8C	1.068
TSC22D4	TSC22 domain family member 4	1.068
TSNARE1	t-SNARE domain containing 1	1.066
MFSD13A	major facilitator superfamily domain containing 13A	1.063
SYP	synaptophysin	1.063
PPP1R3E	protein phosphatase 1 regulatory subunit 3E	1.062
UBL7	ubiquitin like 7	1.062
TRMT112	tRNA methyltransferase subunit 11-2	1.061
PYCR2	pyrroline-5-carboxylate reductase 2	1.057
MAP4K2	mitogen-activated protein kinase kinase kinase kinase 2	1.056
THAP3	THAP domain containing 3	1.056
PRPF6	pre-mRNA processing factor 6	1.055
PUF60	poly(U) binding splicing factor 60	1.051
LZTS2	leucine zipper tumor suppressor 2	1.049
TNFRSF25	TNF receptor superfamily member 25	1.049
RCN2	reticulocalbin 2	1.048
ARHGEF11	Rho guanine nucleotide exchange factor 11	1.042

PLCG1	phospholipase C gamma 1	1.040
ANKRD27	ankyrin repeat domain 27	1.037
PPP2R3B	protein phosphatase 2 regulatory subunit B''beta	1.037
SLX4	SLX4 structure-specific endonuclease subunit	1.037
GPATCH3	G-patch domain containing 3	1.036
PARS2	prolyl-tRNA synthetase 2, mitochondrial	1.036
YEATS2-AS1	YEATS2 antisense RNA 1	1.036
TCFL5	transcription factor like 5	1.034
GNB1L	G protein subunit beta 1 like	1.032
TYSND1	trypsin like peroxisomal matrix peptidase 1	1.032
COMMD5	COMM domain containing 5	1.027
GNPAT	glyceronephosphate O-acyltransferase	1.025
HMGN2P5	high mobility group nucleosomal binding domain 2 pseudogene 5	1.025
SHKBP1	SH3KBP1 binding protein 1	1.025
TMEM250	transmembrane protein 250	1.025
PXMP4	peroxisomal membrane protein 4	1.019
ATP6V1C1	ATPase H+ transporting V1 subunit C1	1.017
FIBP	FGF1 intracellular binding protein	1.016
SNRPN	small nuclear ribonucleoprotein polypeptide N	1.016
ATP6V1E1	ATPase H+ transporting V1 subunit E1	1.015
MARVELD1	MARVEL domain containing 1	1.015
MEN1	menin 1	1.013
CKLF	chemokine like factor	1.012
CROCCP3	CROCC pseudogene 3	1.012
C14orf93	chromosome 14 open reading frame 93	1.010
MYPOP	Myb related transcription factor, partner of profilin	1.010
RNF26	ring finger protein 26	1.009
SIRT7	sirtuin 7	1.008
SRGAP2	SLIT-ROBO Rho GTPase activating protein 2	1.006
ZNF251	zinc finger protein 251	1.003
HSF1	heat shock transcription factor 1	1.002
POP5	POP5 homolog, ribonuclease P/MRP subunit	1.002
CEP112	centrosomal protein 112	1.001
POLR2K	RNA polymerase II, I and III subunit K	1.001
MTMR6	myotubularin related protein 6	-1.007
RBMS1	RNA binding motif single stranded interacting protein 1	-1.008
ZADH2	zinc binding alcohol dehydrogenase domain containing 2	-1.009
ZNF800	zinc finger protein 800	-1.010
CNTLN	centlein	-1.011
PTPN21	protein tyrosine phosphatase non-receptor type 21	-1.011
STRN3	striatin 3	-1.011
ITGA1	integrin subunit alpha 1	-1.012
SLC19A2	solute carrier family 19 member 2	-1.015
C21orf91	chromosome 21 open reading frame 91	-1.016
SLC5A3	solute carrier family 5 member 3	-1.017
DOP1A	DOP1 leucine zipper like protein A	-1.019
TSPYL1	TSPY like 1	-1.020
YTHDC2	YTH domain containing 2	-1.020
DST	dystonin	-1.023
SMAD7	SMAD family member 7	-1.024
C1orf50	chromosome 1 open reading frame 50	-1.030
UTP14C	UTP14C small subunit processome component	-1.031
LGR4	leucine rich repeat containing G protein-coupled receptor 4	-1.032

HECTD1	HECT domain E3 ubiquitin protein ligase 1	-1.033
ZNF160	zinc finger protein 160	-1.035
CRK	CRK proto-oncogene, adaptor protein	-1.036
INPP5B	inositol polyphosphate-5-phosphatase B	-1.036
NFATC2	nuclear factor of activated T cells 2	-1.038
ZNF542P	zinc finger protein 542, pseudogene	-1.040
KAT6A	lysine acetyltransferase 6A	-1.042
SYNE1	spectrin repeat containing nuclear envelope protein 1	-1.042
SH3RF1	SH3 domain containing ring finger 1	-1.045
ATG2B	autophagy related 2B	-1.046
C19orf12	chromosome 19 open reading frame 12	-1.048
RBM15	RNA binding motif protein 15	-1.050
PTS	6-pyruvoyltetrahydropterin synthase	-1.052
ABHD13	abhydrolase domain containing 13	-1.053
CCNB1IP1	cyclin B1 interacting protein 1	-1.056
AOC2	amine oxidase copper containing 2	-1.059
DNAJA1	DnaJ heat shock protein family (Hsp40) member A1	-1.059
RRN3	RRN3 homolog, RNA polymerase I transcription factor	-1.061
ARRB1	arrestin beta 1	-1.062
RP1_95L44		-1.062
ST3GAL6	ST3 beta-galactoside alpha-2,3-sialyltransferase 6	-1.063
BZW1	basic leucine zipper and W2 domains 1	-1.065
ZNF808	zinc finger protein 808	-1.065
USP9X	ubiquitin specific peptidase 9 X-linked	-1.066
L2HGDH	L-2-hydroxyglutarate dehydrogenase	-1.067
MAGI1	membrane associated guanylate kinase, WW and PDZ domain containing 1	-1.068
ZNF586	zinc finger protein 586	-1.069
PPP2CB	protein phosphatase 2 catalytic subunit beta	-1.072
CROCCP2	CROCC pseudogene 2	-1.074
FAM221A	family with sequence similarity 221 member A	-1.074
SEC24D	SEC24 homolog D, COPII coat complex component	-1.076
PVR	PVR cell adhesion molecule	-1.078
DNMBP	dynamin binding protein	-1.080
PEX13	peroxisomal biogenesis factor 13	-1.085
RP11_7F178		-1.089
NMRK1	nicotinamide riboside kinase 1	-1.092
RNFT1	ring finger protein, transmembrane 1	-1.093
SULT1A1	sulfotransferase family 1A member 1	-1.094
NRBF2	nuclear receptor binding factor 2	-1.100
AHI1	Abelson helper integration site 1	-1.104
MPZL3	myelin protein zero like 3	-1.106
ZNF417/ZNF587	zinc finger protein 417	-1.107
USP53	ubiquitin specific peptidase 53	-1.108
CHAMP1	chromosome alignment maintaining phosphoprotein 1	-1.109
RBKS	ribokinase	-1.109
PHKA1	phosphorylase kinase regulatory subunit alpha 1	-1.110
L3MBTL4	L3MBTL histone methyl-lysine binding protein 4	-1.112
ZNF395	zinc finger protein 395	-1.112
MT-ND4L	NADH dehydrogenase subunit 4L	-1.114
ZNF274	zinc finger protein 274	-1.114
CPNE8	copine 8	-1.117
EHBP1	EH domain binding protein 1	-1.118
INTS6	integrator complex subunit 6	-1.118

HADHB	hydroxyacyl-CoA dehydrogenase trifunctional multienzyme complex subunit beta	-1.120
INSR	insulin receptor	-1.122
USO1	USO1 vesicle transport factor	-1.124
AFF1	AF4/FMR2 family member 1	-1.126
DNAJC27	DnaJ heat shock protein family (Hsp40) member C27	-1.126
ENTPD7	ectonucleoside triphosphate diphosphohydrolase 7	-1.126
INTS10	integrator complex subunit 10	-1.126
CDADC1	cytidine and dCMP deaminase domain containing 1	-1.128
RAPGEF2	Rap guanine nucleotide exchange factor 2	-1.128
PPFIBP1	PPFIA binding protein 1	-1.129
WDFY2	WD repeat and FYVE domain containing 2	-1.129
FAM167B	family with sequence similarity 167 member B	-1.136
SEC23A	SEC23 homolog A, COPII coat complex component	-1.136
FRMD4B	FERM domain containing 4B	-1.137
RP1_151F172		-1.140
CHD1	chromodomain helicase DNA binding protein 1	-1.141
KLHL36	kelch like family member 36	-1.142
RP11_110I111		-1.142
JAK2	Janus kinase 2	-1.148
GCKR	glucokinase regulator	-1.151
PXMP2	peroxisomal membrane protein 2	-1.153
DDX19B	DEAD-box helicase 19B	-1.154
NOSTRIN	nitric oxide synthase trafficking	-1.158
NCOA4	nuclear receptor coactivator 4	-1.159
ATP11C	ATPase phospholipid transporting 11C	-1.163
LMO7	LIM domain 7	-1.163
SGPP1	sphingosine-1-phosphate phosphatase 1	-1.163
ZNF121	zinc finger protein 121	-1.163
LOC101927752	uncharacterized LOC101927752	-1.168
ZNF844	zinc finger protein 844	-1.169
HERC3	HECT and RLD domain containing E3 ubiquitin protein ligase 3	-1.170
BZW1P2	basic leucine zipper and W2 domains 1 pseudogene 2	-1.174
KDM7A	lysine demethylase 7A	-1.176
LMAN1	lectin, mannose binding 1	-1.176
RWDD2A	RWD domain containing 2A	-1.176
ZNF805	zinc finger protein 805	-1.176
HIPK3	homeodomain interacting protein kinase 3	-1.177
MAP2K1	mitogen-activated protein kinase kinase 1	-1.181
AL035078.4		-1.182
TFPI	tissue factor pathway inhibitor	-1.183
MAP4K3-DT	MAP4K3 divergent transcript	-1.184
ZNF649	zinc finger protein 649	-1.187
DENND4A	DENN domain containing 4A	-1.188
SHB	SH2 domain containing adaptor protein B	-1.188
ANKS4B	ankyrin repeat and sterile alpha motif domain containing 4B	-1.189
RMDN2	regulator of microtubule dynamics 2	-1.192
RPH3AL	rabphilin 3A like (without C2 domains)	-1.193
SMPDL3A	sphingomyelin phosphodiesterase acid like 3A	-1.195
RHOU	ras homolog family member U	-1.196
BMERB1	bMERB domain containing 1	-1.199
COG3	component of oligomeric golgi complex 3	-1.200
FBXO21	F-box protein 21	-1.203
OTUD4	OTU deubiquitinase 4	-1.207

FEM1C	fem-1 homolog C	-1.208
DMRTA1	DMRT like family A1	-1.210
RANBP6	RAN binding protein 6	-1.210
ZCCHC2	zinc finger CCHC-type containing 2	-1.211
ALG11	ALG11 alpha-1,2-mannosyltransferase	-1.212
ACADS	acyl-CoA dehydrogenase short chain	-1.217
FZD5	frizzled class receptor 5	-1.217
STAT3	signal transducer and activator of transcription 3	-1.217
RHPN2	rhophilin Rho GTPase binding protein 2	-1.218
AKAP13	A-kinase anchoring protein 13	-1.221
CFL2	cofilin 2	-1.221
PLA2G12A	phospholipase A2 group XIIA	-1.222
ABCD3	ATP binding cassette subfamily D member 3	-1.223
ABHD5	abhydrolase domain containing 5, lysophosphatidic acid acyltransferase	-1.224
SELENOI	selenoprotein I	-1.226
SIK3	SIK family kinase 3	-1.228
DDX3X	DEAD-box helicase 3 X-linked	-1.231
POLR1E	RNA polymerase I subunit E	-1.240
CDK3	cyclin dependent kinase 3	-1.241
IFNAR1	interferon alpha and beta receptor subunit 1	-1.245
SEC24A	SEC24 homolog A, COPII coat complex component	-1.246
AC0920661		-1.247
BDH2	3-hydroxybutyrate dehydrogenase 2	-1.247
DAPK1	death associated protein kinase 1	-1.247
LINC00242	long intergenic non-protein coding RNA 242	-1.247
PGM1	phosphoglucomutase 1	-1.248
MIDN	midnolin	-1.249
OSBPL11	oxysterol binding protein like 11	-1.250
TBC1D8B	TBC1 domain family member 8B	-1.250
RAB33B	RAB33B, member RAS oncogene family	-1.253
TMEM131L	transmembrane 131 like	-1.253
NAXD	NAD(P)HX dehydratase	-1.254
ZBTB43	zinc finger and BTB domain containing 43	-1.259
ZCCHC24	zinc finger CCHC-type containing 24	-1.259
SLC16A1	solute carrier family 16 member 1	-1.262
CNOT6L	CCR4-NOT transcription complex subunit 6 like	-1.265
DBT	dihydrolipoamide branched chain transacylase E2	-1.266
TRAM2	translocation associated membrane protein 2	-1.267
PHLDB2	pleckstrin homology like domain family B member 2	-1.268
RP11_373L241		-1.268
ELF1	E74 like ETS transcription factor 1	-1.270
HDAC6	histone deacetylase 6	-1.273
INPP1	inositol polyphosphate-1-phosphatase	-1.273
TTPAL	alpha tocopherol transfer protein like	-1.273
WWC2	WW and C2 domain containing 2	-1.276
C1RL	complement C1r subcomponent like	-1.280
SH3YL1	SH3 and SYLF domain containing 1	-1.284
IQGAP2	IQ motif containing GTPase activating protein 2	-1.289
MLYCD	malonyl-CoA decarboxylase	-1.289
SOCS6	suppressor of cytokine signaling 6	-1.289
F11R	F11 receptor	-1.291
NR3C2	nuclear receptor subfamily 3 group C member 2	-1.291
MTCO1P12	MT-CO1 pseudogene 12	-1.292

C17orf100	chromosome 17 open reading frame 100	-1.296
GPRASP1	G protein-coupled receptor associated sorting protein 1	-1.298
MRPS31P4	mitochondrial ribosomal protein S31 pseudogene 4	-1.303
KIAA1671	KIAA1671	-1.306
FNDC3A	fibronectin type III domain containing 3A	-1.310
REPS2	RALBP1 associated Eps domain containing 2	-1.311
IFFO2	intermediate filament family orphan 2	-1.313
ADK	adenosine kinase	-1.316
MROH8	maestro heat like repeat family member 8	-1.316
TLR3	toll like receptor 3	-1.316
RP11_529K13		-1.317
SEC24B	SEC24 homolog B, COPII coat complex component	-1.317
MYD88	MYD88 innate immune signal transduction adaptor	-1.318
ALDH1B1	aldehyde dehydrogenase 1 family member B1	-1.320
LRRK2	leucine rich repeat kinase 2	-1.327
WEE1	WEE1 G2 checkpoint kinase	-1.327
MAP7	microtubule associated protein 7	-1.328
SLC31A1	solute carrier family 31 member 1	-1.329
EIF2AK3	eukaryotic translation initiation factor 2 alpha kinase 3	-1.333
TTC39C	tetratricopeptide repeat domain 39C	-1.335
RP11_96D111		-1.337
FANCC	FA complementation group C	-1.340
HPS5	HPS5 biogenesis of lysosomal organelles complex 2 subunit 2	-1.349
PSAT1	phosphoserine aminotransferase 1	-1.350
PUS10	pseudouridine synthase 10	-1.350
CTC_444N2411		-1.353
STARD4	StAR related lipid transfer domain containing 4	-1.359
RAB27A	RAB27A, member RAS oncogene family	-1.362
MT-CO1	cytochrome c oxidase subunit I	-1.363
TMEM30BP1	TMEM30B pseudogene 1	-1.364
MTARC1	mitochondrial amidoxime reducing component 1	-1.368
SHPK	sedoheptulokinase	-1.368
ONECUT1	one cut homeobox 1	-1.370
PLPBP	pyridoxal phosphate binding protein	-1.370
SLC31A2	solute carrier family 31 member 2	-1.371
XBP1	X-box binding protein 1	-1.373
SOWAHC	sosondowah ankyrin repeat domain family member C	-1.374
IVD	isovaleryl-CoA dehydrogenase	-1.375
TIPARP	TCDD inducible poly(ADP-ribose) polymerase	-1.376
MAP2K3	mitogen-activated protein kinase kinase 3	-1.378
FAM13A	family with sequence similarity 13 member A	-1.383
SPATA13	spermatogenesis associated 13	-1.385
PLK2	polo like kinase 2	-1.387
DNAJC25	DnaJ heat shock protein family (Hsp40) member C25	-1.388
HSPA4L	heat shock protein family A (Hsp70) member 4 like	-1.390
CCT6B	chaperonin containing TCP1 subunit 6B	-1.391
PID1	phosphotyrosine interaction domain containing 1	-1.399
CTBS	chitobiase	-1.403
C4A-AS1		-1.404
C4B-AS1		-1.404
SUCLG2-AS1	SUCLG2 antisense RNA 1 (head to head)	-1.404
CFAP251	cilia and flagella associated protein 251	-1.407
PNPLA7	patatin like phospholipase domain containing 7	-1.408

HIVEP2	HIVEP zinc finger 2	-1.411
PGM3	phosphoglucomutase 3	-1.412
TRIM35	tripartite motif containing 35	-1.412
CTB_50E145		-1.413
F8	coagulation factor VIII	-1.413
PLEKHA7	pleckstrin homology domain containing A7	-1.413
PLPP6	phospholipid phosphatase 6	-1.413
ABCA1	ATP binding cassette subfamily A member 1	-1.414
PER2	period circadian regulator 2	-1.414
KLHL2	kelch like family member 2	-1.415
PPP2R1B	protein phosphatase 2 scaffold subunit Abeta	-1.419
HIVEP1	HIVEP zinc finger 1	-1.421
ACADM	acyl-CoA dehydrogenase medium chain	-1.422
FAM229B	family with sequence similarity 229 member B	-1.422
CCDC186	coiled-coil domain containing 186	-1.424
PDXDC2P	pyridoxal dependent decarboxylase domain containing 2, pseudogene	-1.428
RP1_253P74		-1.429
SMOC1	SPARC related modular calcium binding 1	-1.431
DHRS1	dehydrogenase/reductase 1	-1.432
ZFAND5	zinc finger AN1-type containing 5	-1.432
GALNT3	polypeptide N-acetylgalactosaminyltransferase 3	-1.434
RP3_468K186		-1.435
DCUN1D3	defective in cullin neddylation 1 domain containing 3	-1.436
GLIS3	GLIS family zinc finger 3	-1.436
FERMT2	FERM domain containing kindlin 2	-1.438
MYO1B	myosin IB	-1.438
SLC25A20	solute carrier family 25 member 20	-1.438
NDFIP2	Nedd4 family interacting protein 2	-1.439
B4GALT1-AS1	B4GALT1 antisense RNA 1	-1.440
EIF5	eukaryotic translation initiation factor 5	-1.444
TMEM220-AS1	TMEM220 antisense RNA 1	-1.444
ARHGEF26	Rho guanine nucleotide exchange factor 26	-1.449
MAFK	MAF bZIP transcription factor K	-1.450
C9orf72	C9orf72-SMCR8 complex subunit	-1.456
SPRYD4	SPRY domain containing 4	-1.457
TTC39B	tetratricopeptide repeat domain 39B	-1.458
PPP2R2A	protein phosphatase 2 regulatory subunit Balpha	-1.461
TNFRSF10B	TNF receptor superfamily member 10b	-1.462
APOL6	apolipoprotein L6	-1.463
DOCK5	dedicator of cytokinesis 5	-1.469
IRF2BPL	interferon regulatory factor 2 binding protein like	-1.472
RAPGEF5	Rap guanine nucleotide exchange factor 5	-1.481
C2orf88	chromosome 2 open reading frame 88	-1.485
EDEM1	ER degradation enhancing alpha-mannosidase like protein 1	-1.487
IPCEF1	interaction protein for cytohesin exchange factors 1	-1.487
MAP1LC3A	microtubule associated protein 1 light chain 3 alpha	-1.488
POR	cytochrome p450 oxidoreductase	-1.489
ATXN1-AS1	ATXN1 antisense RNA 1	-1.491
RGPD4 (includes others)	RANBP2 like and GRIP domain containing 5	-1.493
ACER2	alkaline ceramidase 2	-1.495
ACAD11	acyl-CoA dehydrogenase family member 11	-1.496
ABHD18	abhydrolase domain containing 18	-1.498

DLG2	discs large MAGUK scaffold protein 2	-1.498
RP11_325K42		-1.503
PROX1	prospero homeobox 1	-1.508
PROX1-AS1	PROX1 antisense RNA 1	-1.508
RDH5	retinol dehydrogenase 5	-1.508
TJP2	tight junction protein 2	-1.509
CLN8	CLN8 transmembrane ER and ERGIC protein	-1.510
SLC20A1	solute carrier family 20 member 1	-1.511
LAG3	lymphocyte activating 3	-1.515
SH3BGRL2	SH3 domain binding glutamate rich protein like 2	-1.517
GNG12-AS1	GNG12, DIRAS3 and WLS antisense RNA 1	-1.519
RP11_93B149		-1.523
ATP7B	ATPase copper transporting beta	-1.524
PTPRB	protein tyrosine phosphatase receptor type B	-1.525
CLDN1	claudin 1	-1.530
CREM	cAMP responsive element modulator	-1.539
C17orf107	chromosome 17 open reading frame 107	-1.540
COBL	cordon-bleu WH2 repeat protein	-1.540
CHP1	calcineurin like EF-hand protein 1	-1.541
CRYBG1	crystallin beta-gamma domain containing 1	-1.542
IRF1	interferon regulatory factor 1	-1.542
TPST1	tyrosylprotein sulfotransferase 1	-1.544
MT-ND5	NADH dehydrogenase subunit 5	-1.551
AC0094042		-1.553
AVPI1	arginine vasopressin induced 1	-1.553
PDE3B	phosphodiesterase 3B	-1.554
RP11_75C106		-1.555
IRF8	interferon regulatory factor 8	-1.561
TIAM1	TIAM Rac1 associated GEF 1	-1.561
DOCK8-AS1	DOCK8 antisense RNA 1	-1.562
FNIP2	folliculin interacting protein 2	-1.562
USP38	ubiquitin specific peptidase 38	-1.564
GPHN	gephyrin	-1.569
PSD4	pleckstrin and Sec7 domain containing 4	-1.569
REL	REL proto-oncogene, NF-kB subunit	-1.571
ARL4D	ADP ribosylation factor like GTPase 4D	-1.575
NAP1L5	nucleosome assembly protein 1 like 5	-1.576
PRKAG2	protein kinase AMP-activated non-catalytic subunit gamma 2	-1.577
MYCT1	MYC target 1	-1.581
AMBP	alpha-1-microglobulin/bikunin precursor	-1.583
IFNLR1	interferon lambda receptor 1	-1.586
EPOR	erythropoietin receptor	-1.587
STAT4	signal transducer and activator of transcription 4	-1.588
PBLD	phenazine biosynthesis like protein domain containing	-1.590
SLC35D1	solute carrier family 35 member D1	-1.590
DNAJC12	DnaJ heat shock protein family (Hsp40) member C12	-1.591
TMEM86B	transmembrane protein 86B	-1.593
SLA2	Src like adaptor 2	-1.598
PEX11G	peroxisomal biogenesis factor 11 gamma	-1.599
RPL17-C18orf32	RPL17-C18orf32 readthrough	-1.601
SALL1	spalt like transcription factor 1	-1.602
FEZ1	fasciculation and elongation protein zeta 1	-1.605
AGMO	alkylglycerol monooxygenase	-1.607

LATS2	large tumor suppressor kinase 2	-1.609
FXN	frataxin	-1.610
ABHD15	abhydrolase domain containing 15	-1.613
RBMS3	RNA binding motif single stranded interacting protein 3	-1.613
KYNU	kynureninase	-1.615
PRRG4	proline rich and Gla domain 4	-1.615
TGDS	TDP-glucose 4,6-dehydratase	-1.615
ZSWIM6	zinc finger SWIM-type containing 6	-1.620
PPIEL	peptidylprolyl isomerase E like pseudogene	-1.622
EPB41L4A	erythrocyte membrane protein band 4.1 like 4A	-1.623
NTN4	netrin 4	-1.624
LONP2	lon peptidase 2, peroxisomal	-1.627
C16orf96	chromosome 16 open reading frame 96	-1.628
NONOP2	non-POU domain containing, octamer-binding pseudogene 2	-1.634
ELL2P1	elongation factor for RNA polymerase II 2 pseudogene 1	-1.637
TECTA	tectorin alpha	-1.637
ERN1	endoplasmic reticulum to nucleus signaling 1	-1.638
USP12	ubiquitin specific peptidase 12	-1.638
ACO1	aconitase 1	-1.639
DUSP16	dual specificity phosphatase 16	-1.639
CCR1	C-C motif chemokine receptor 1	-1.641
RP11_338I211		-1.643
MTRNR2L12	MT-RNR2 like 12	-1.645
TSPYL2	TSPY like 2	-1.645
FRMD6	FERM domain containing 6	-1.649
NSUN6	NOP2/Sun RNA methyltransferase 6	-1.649
MCL1	MCL1 apoptosis regulator, BCL2 family member	-1.652
RP11_629B115		-1.652
SLC9B2	solute carrier family 9 member B2	-1.653
ADAP1	ArfGAP with dual PH domains 1	-1.656
CDNF	cerebral dopamine neurotrophic factor	-1.657
GATA6	GATA binding protein 6	-1.658
CBLC	Cbl proto-oncogene C	-1.659
OLFML3	olfactomedin like 3	-1.660
PIK3R1	phosphoinositide-3-kinase regulatory subunit 1	-1.661
RP11_544M2213		-1.665
AGL	amylo-alpha-1, 6-glucosidase, 4-alpha-glucanotransferase	-1.666
SORBS1	sorbin and SH3 domain containing 1	-1.667
ARL5B	ADP ribosylation factor like GTPase 5B	-1.671
ERLIN1	ER lipid raft associated 1	-1.671
SPRY2	sprouty RTK signaling antagonist 2	-1.674
GLRX	glutaredoxin	-1.675
TP53INP2	tumor protein p53 inducible nuclear protein 2	-1.677
EPAS1	endothelial PAS domain protein 1	-1.680
KLF11	Kruppel like factor 11	-1.683
PRF1	perforin 1	-1.683
TMOD1	tropomodulin 1	-1.684
PLIN5	perilipin 5	-1.685
EGFR	epidermal growth factor receptor	-1.690
SLCO2B1	solute carrier organic anion transporter family member 2B1	-1.691
UAP1	UDP-N-acetylglucosamine pyrophosphorylase 1	-1.693
CAVIN2	caveolae associated protein 2	-1.694
WWC1	WW and C2 domain containing 1	-1.694

ACACB	acetyl-CoA carboxylase beta	-1.698
CCDC68	coiled-coil domain containing 68	-1.700
MMAA	metabolism of cobalamin associated A	-1.700
BCKDHB	branched chain keto acid dehydrogenase E1 subunit beta	-1.701
TMEM220	transmembrane protein 220	-1.701
ABHD2	abhydrolase domain containing 2, acylglycerol lipase	-1.705
GNPNAT1	glucosamine-phosphate N-acetyltransferase 1	-1.705
MTCO1P2	MT-CO1 pseudogene 2	-1.706
DUSP10	dual specificity phosphatase 10	-1.710
KIAA0040	KIAA0040	-1.715
ANTXR2	ANTXR cell adhesion molecule 2	-1.718
LINC02637	long intergenic non-protein coding RNA 2637	-1.719
SLC25A25	solute carrier family 25 member 25	-1.723
VMO1	vitelline membrane outer layer 1 homolog	-1.725
DMD	dystrophin	-1.729
PEMT	phosphatidylethanolamine N-methyltransferase	-1.731
LIPC	lipase C, hepatic type	-1.733
GPT	glutamic--pyruvic transaminase	-1.735
RP11_42O153		-1.735
LNX2	ligand of numb-protein X 2	-1.741
NDEL1	nudE neurodevelopment protein 1 like 1	-1.741
LINC00261	long intergenic non-protein coding RNA 261	-1.748
CHRNE	cholinergic receptor nicotinic epsilon subunit	-1.752
IFITM10	interferon induced transmembrane protein 10	-1.756
TENT5C	terminal nucleotidyltransferase 5C	-1.762
KDM6B	lysine demethylase 6B	-1.771
ABCA9	ATP binding cassette subfamily A member 9	-1.774
PRSS53	serine protease 53	-1.777
NAAA	N-acylethanolamine acid amidase	-1.778
OR7E14P	olfactory receptor family 7 subfamily E member 14 pseudogene	-1.779
B4GALT1	beta-1,4-galactosyltransferase 1	-1.783
MIDEAS	mitotic deacetylase associated SANT domain protein	-1.783
RASSF6	Ras association domain family member 6	-1.783
SLC41A2	solute carrier family 41 member 2	-1.786
ACSM3	acyl-CoA synthetase medium chain family member 3	-1.788
CFLAR-AS1	CFLAR antisense RNA 1	-1.788
TGFBR3	transforming growth factor beta receptor 3	-1.788
RAB20	RAB20, member RAS oncogene family	-1.790
C11orf54	chromosome 11 open reading frame 54	-1.793
LARP1B	La ribonucleoprotein 1B	-1.796
HOOK1	hook microtubule tethering protein 1	-1.797
BCL3	BCL3 transcription coactivator	-1.798
ACAA2	acetyl-CoA acyltransferase 2	-1.801
GFOD1	glucose-fructose oxidoreductase domain containing 1	-1.804
NDRG2	NDRG family member 2	-1.807
ZC2HC1C	zinc finger C2HC-type containing 1C	-1.809
ARAP2	ArfGAP with RhoGAP domain, ankyrin repeat and PH domain 2	-1.820
MTHFD1	methylenetetrahydrofolate dehydrogenase, cyclohydrolase and formyltetrahydrofolate synthetase 1	-1.825
TLR4	toll like receptor 4	-1.832
IL4R	interleukin 4 receptor	-1.838
ACAA1	acetyl-CoA acyltransferase 1	-1.850
SYDE2	synapse defective Rho GTPase homolog 2	-1.850

MT-RNR2	l-rRNA	-1.858
PLSCR4	phospholipid scramblase 4	-1.863
FAM149A	family with sequence similarity 149 member A	-1.865
SLC4A4	solute carrier family 4 member 4	-1.865
FYB2	FYN binding protein 2	-1.866
GZMB	granzyme B	-1.869
CTD_2373N43		-1.872
SAMD4A	sterile alpha motif domain containing 4A	-1.873
RP11-606E8.2		-1.876
GCDH	glutaryl-CoA dehydrogenase	-1.877
HGD	homogentisate 1,2-dioxygenase	-1.877
CPP	ceruloplasmin pseudogene	-1.879
GABARAPL1	GABA type A receptor associated protein like 1	-1.879
IGFBP3	insulin like growth factor binding protein 3	-1.879
ELL2	elongation factor for RNA polymerase II 2	-1.880
MMUT	methylmalonyl-CoA mutase	-1.880
KAZN	kazrin, periplakin interacting protein	-1.890
RTL5	retrotransposon Gag like 5	-1.890
IL6ST	interleukin 6 cytokine family signal transducer	-1.892
MYO10	myosin X	-1.892
PDLIM5	PDZ and LIM domain 5	-1.894
IL2RB	interleukin 2 receptor subunit beta	-1.896
IDNK	IDNK gluconokinase	-1.902
CYP2J2	cytochrome P450 family 2 subfamily J member 2	-1.908
PXDC1	PX domain containing 1	-1.909
MPC1	mitochondrial pyruvate carrier 1	-1.911
SPTBN2	spectrin beta, non-erythrocytic 2	-1.914
PCDH19	protocadherin 19	-1.916
GJB2	gap junction protein beta 2	-1.917
DEPDC7	DEP domain containing 7	-1.919
NAALADL2	N-acetylated alpha-linked acidic dipeptidase like 2	-1.920
TBXA2R	thromboxane A2 receptor	-1.920
SLC23A2	solute carrier family 23 member 2	-1.924
GBP1	guanylate binding protein 1	-1.930
ITGA9	integrin subunit alpha 9	-1.940
PLIN4	perilipin 4	-1.942
PTP4A1	protein tyrosine phosphatase 4A1	-1.947
CISH	cytokine inducible SH2 containing protein	-1.949
SMPD3	sphingomyelin phosphodiesterase 3	-1.950
GNE	glucosamine (UDP-N-acetyl)-2-epimerase/N-acetylmannosamine kinase	-1.951
TCHH	trichohyalin	-1.955
FAAH2	fatty acid amide hydrolase 2	-1.958
RP11_449P151		-1.958
EOMES	eomesodermin	-1.959
RP11_463O125		-1.959
RP11_437L71		-1.970
ACMSD	aminocarboxymuconate semialdehyde decarboxylase	-1.975
TUBBP10	tubulin beta class I pseudogene 10	-1.975
CD302	CD302 molecule	-1.978
MYO1E	myosin IE	-1.978
SLC19A3	solute carrier family 19 member 3	-1.983
TUBE1	tubulin epsilon 1	-1.983
HAAO	3-hydroxyanthranilate 3,4-dioxygenase	-1.985

CP	ceruloplasmin	-1.987
ACOX1	acyl-CoA oxidase 1	-1.988
CPED1	cadherin like and PC-esterase domain containing 1	-1.994
AL358113.1		-1.997
DYNLT5	dynein light chain Tctex-type family member 5	-1.997
CROCC2	ciliary rootlet coiled-coil, rootletin family member 2	-2.000
NFKBIZ	NFKB inhibitor zeta	-2.000
YPEL2	yippee like 2	-2.002
CFD	complement factor D	-2.005
ORM1	orosomucoid 1	-2.007
RXFP1	relaxin family peptide receptor 1	-2.020
CLEC12A	C-type lectin domain family 12 member A	-2.021
CST7	cystatin F	-2.021
PDCD1LG2	programmed cell death 1 ligand 2	-2.025
PDE2A	phosphodiesterase 2A	-2.026
LONRF3	LON peptidase N-terminal domain and ring finger 3	-2.027
EPHA2	EPH receptor A2	-2.028
NAV2	neuron navigator 2	-2.029
P2RY10	P2Y receptor family member 10	-2.029
FITM1	fat storage inducing transmembrane protein 1	-2.030
LOC101927793	uncharacterized LOC101927793	-2.033
CTD_3203P23		-2.038
FOSL2	FOS like 2, AP-1 transcription factor subunit	-2.038
GOT1	glutamic-oxaloacetic transaminase 1	-2.043
GNA14	G protein subunit alpha 14	-2.044
STOM	stomatin	-2.045
TLCD4	TLC domain containing 4	-2.048
SERPINB8	serpin family B member 8	-2.055
CD160	CD160 molecule	-2.057
ID2-AS1	ID2 antisense RNA 1	-2.060
ORM2	orosomucoid 2	-2.062
SLC38A2	solute carrier family 38 member 2	-2.063
CLTRN	collectrin, amino acid transport regulator	-2.064
LPIN2	lipin 2	-2.068
IL33	interleukin 33	-2.069
LILRA2	leukocyte immunoglobulin like receptor A2	-2.077
SRD5A1	steroid 5 alpha-reductase 1	-2.087
RP11_130L82		-2.088
RIPK4	receptor interacting serine/threonine kinase 4	-2.089
GPR180	G protein-coupled receptor 180	-2.090
ALAS1	5'-aminolevulinate synthase 1	-2.093
STX11	syntaxin 11	-2.095
CPEB3	cytoplasmic polyadenylation element binding protein 3	-2.097
ABLIM3	actin binding LIM protein family member 3	-2.098
ETFDH	electron transfer flavoprotein dehydrogenase	-2.106
CCDC71L	coiled-coil domain containing 71 like	-2.112
DUSP5	dual specificity phosphatase 5	-2.113
RP11_359K184		-2.123
MPEG1	macrophage expressed 1	-2.129
HS3ST3A1	heparan sulfate-glucosamine 3-sulfotransferase 3A1	-2.134
KCNA3	potassium voltage-gated channel subfamily A member 3	-2.134
MCC	MCC regulator of WNT signaling pathway	-2.154
SDCBP2	syndecan binding protein 2	-2.167

PLIN2	perilipin 2	-2.168
CTC_526N191		-2.172
AC009093.10		-2.178
CCL14	C-C motif chemokine ligand 14	-2.178
CD38	CD38 molecule	-2.178
ZNF638-IT1		-2.186
STEAP3-AS1	STEAP3 antisense RNA 1	-2.192
DHODH	dihydroorotate dehydrogenase (quinone)	-2.194
LOC105375304	uncharacterized LOC105375304	-2.196
NAMPT	nicotinamide phosphoribosyltransferase	-2.205
ADM	adrenomedullin	-2.207
PLPP3	phospholipid phosphatase 3	-2.209
IL18RAP	interleukin 18 receptor accessory protein	-2.221
KLHL15	kelch like family member 15	-2.234
SARDH	sarcosine dehydrogenase	-2.235
GPT2	glutamic--pyruvic transaminase 2	-2.236
PSD3	pleckstrin and Sec7 domain containing 3	-2.241
ANKRD53	ankyrin repeat domain 53	-2.243
EHD3	EH domain containing 3	-2.244
PLGLB1/PLGLB2	plasminogen like B2	-2.245
LY6E	lymphocyte antigen 6 family member E	-2.246
GK-AS1	GK antisense RNA 1	-2.248
FCER2	Fc fragment of IgE receptor II	-2.249
FLT3	fms related receptor tyrosine kinase 3	-2.249
SCIMP	SLP adaptor and CSK interacting membrane protein	-2.250
TGFB3	transforming growth factor beta 3	-2.253
TPPP2	tubulin polymerization promoting protein family member 2	-2.253
AC00507714		-2.257
LRAT	lecithin retinol acyltransferase	-2.257
CYFIP2	cytoplasmic FMR1 interacting protein 2	-2.258
RCAN1	regulator of calcineurin 1	-2.258
BRINP1	BMP/retinoic acid inducible neural specific 1	-2.263
CLIC6	chloride intracellular channel 6	-2.270
CDC37L1	cell division cycle 37 like 1	-2.272
LTK	leukocyte receptor tyrosine kinase	-2.275
SYTL3	synaptotagmin like 3	-2.276
TMEM30B	transmembrane protein 30B	-2.276
RP11-331G2.7		-2.277
SH2D1B	SH2 domain containing 1B	-2.280
CD8A	CD8a molecule	-2.281
NFIL3	nuclear factor, interleukin 3 regulated	-2.283
ADGRG6	adhesion G protein-coupled receptor G6	-2.284
CHORDC1P4	CHORDC1 pseudogene 4	-2.292
TACSTD2	tumor associated calcium signal transducer 2	-2.297
KLRC4-KLRK1/KLRK1	killer cell lectin like receptor K1	-2.299
EPB41L4B	erythrocyte membrane protein band 4.1 like 4B	-2.305
HMOX1	heme oxygenase 1	-2.305
PTPRT	protein tyrosine phosphatase receptor type T	-2.305
CTB_50L1714		-2.307
RCL1	RNA terminal phosphate cyclase like 1	-2.307
IRF4	interferon regulatory factor 4	-2.311
TRPV4	transient receptor potential cation channel subfamily V member 4	-2.317

AQP7	aquaporin 7	-2.323
ZBTB21	zinc finger and BTB domain containing 21	-2.328
IL18R1	interleukin 18 receptor 1	-2.330
DLC1	DLC1 Rho GTPase activating protein	-2.333
DBH-AS1	DBH antisense RNA 1	-2.336
CPT1A	carnitine palmitoyltransferase 1A	-2.338
TMPRSS2	transmembrane serine protease 2	-2.339
FOLH1	folate hydrolase 1	-2.340
SDC4	syndecan 4	-2.346
PANK1	pantothenate kinase 1	-2.348
LRRN3	leucine rich repeat neuronal 3	-2.349
CYP39A1	cytochrome P450 family 39 subfamily A member 1	-2.351
PFKFB3	6-phosphofructo-2-kinase/fructose-2,6-biphosphatase 3	-2.351
CCDC38	coiled-coil domain containing 38	-2.355
CIDEB	cell death inducing DFFA like effector b	-2.356
C5AR1	complement C5a receptor 1	-2.359
TRIB1	tribbles pseudokinase 1	-2.367
RP11_242C192		-2.384
HOGA1	4-hydroxy-2-oxoglutarate aldolase 1	-2.386
ZNF831	zinc finger protein 831	-2.386
LRRN1	leucine rich repeat neuronal 1	-2.387
SLC16A2	solute carrier family 16 member 2	-2.394
SGK1	serum/glucocorticoid regulated kinase 1	-2.395
ALDH8A1	aldehyde dehydrogenase 8 family member A1	-2.399
PRRT1B	proline rich transmembrane protein 1B	-2.399
LIPG	lipase G, endothelial type	-2.402
IGFBP1	insulin like growth factor binding protein 1	-2.403
CMYA5	cardiomyopathy associated 5	-2.410
CYP4V2	cytochrome P450 family 4 subfamily V member 2	-2.423
MBNL2	muscleblind like splicing regulator 2	-2.425
AKAP12	A-kinase anchoring protein 12	-2.428
GLDCP1	glycine decarboxylase pseudogene 1	-2.429
PNRC1	proline rich nuclear receptor coactivator 1	-2.432
GADD45G	growth arrest and DNA damage inducible gamma	-2.433
FGL2	fibrinogen like 2	-2.435
SLC39A14	solute carrier family 39 member 14	-2.436
TNFRSF10D	TNF receptor superfamily member 10d	-2.438
RASGEF1B	RasGEF domain family member 1B	-2.440
MIP	major intrinsic protein of lens fiber	-2.442
GRAMD1C	GRAM domain containing 1C	-2.449
ID2	inhibitor of DNA binding 2	-2.451
ETS2	ETS proto-oncogene 2, transcription factor	-2.452
SH2D1A	SH2 domain containing 1A	-2.454
PKHD1	PKHD1 ciliary IPT domain containing fibrocystin/polyductin	-2.463
TMEM26	transmembrane protein 26	-2.464
LPAL2	lipoprotein(a) like 2, pseudogene	-2.468
GLDC	glycine decarboxylase	-2.480
KLKB1	kallikrein B1	-2.481
ADAMTS1	ADAM metallopeptidase with thrombospondin type 1 motif 1	-2.483
GAREM1	GRB2 associated regulator of MAPK1 subtype 1	-2.487
DIP2C-AS1	DIP2C antisense RNA 1	-2.490
F11	coagulation factor XI	-2.491
C8A	complement C8 alpha chain	-2.494

TGFA	transforming growth factor alpha	-2.495
BACH2	BTB domain and CNC homolog 2	-2.499
GRHL1	grainyhead like transcription factor 1	-2.510
GTF2IP7	general transcription factor IIi pseudogene 7	-2.513
PITPNM3	PITPNM family member 3	-2.514
SLC1A1	solute carrier family 1 member 1	-2.517
FCRL1	Fc receptor like 1	-2.523
LY6E-DT	LY6E divergent transcript	-2.528
RELN	reelin	-2.535
CPN1	carboxypeptidase N subunit 1	-2.545
KRTCAP3	keratinocyte associated protein 3	-2.552
KLRD1	killer cell lectin like receptor D1	-2.560
RND3	Rho family GTPase 3	-2.563
DUSP1	dual specificity phosphatase 1	-2.565
CRTAM	cytotoxic and regulatory T cell molecule	-2.577
MIR22HG	MIR22 host gene	-2.577
PALM2AKAP2	PALM2 and AKAP2 fusion	-2.577
AASS	aminoadipate-semialdehyde synthase	-2.580
MFAP3L	microfibril associated protein 3 like	-2.581
ZFP36	ZFP36 ring finger protein	-2.583
KBTBD11	kelch repeat and BTB domain containing 11	-2.584
ANO1	anoctamin 1	-2.586
KCNE1	potassium voltage-gated channel subfamily E regulatory subunit 1	-2.607
ID1	inhibitor of DNA binding 1, HLH protein	-2.616
RNF152	ring finger protein 152	-2.617
CH507_42P116		-2.618
TCP10L	t-complex 10 like	-2.624
CTH	cystathionine gamma-lyase	-2.625
GK	glycerol kinase	-2.627
SGMS2	sphingomyelin synthase 2	-2.630
CTNNA3	catenin alpha 3	-2.631
BICDL2	BICD family like cargo adaptor 2	-2.632
PPARGC1A	PPARG coactivator 1 alpha	-2.634
VNN3	vanin 3	-2.635
COLEC11	collectin subfamily member 11	-2.638
HS3ST3B1	heparan sulfate-glucosamine 3-sulfotransferase 3B1	-2.647
ATP13A4	ATPase 13A4	-2.658
PON3	paraoxonase 3	-2.659
GDF7	growth differentiation factor 7	-2.666
ANGPTL6	angiopoietin like 6	-2.676
DTHD1	death domain containing 1	-2.681
GPAT3	glycerol-3-phosphate acyltransferase 3	-2.687
IL1RAP	interleukin 1 receptor accessory protein	-2.687
ATOH8	atonal bHLH transcription factor 8	-2.688
SIGLEC11	sialic acid binding Ig like lectin 11	-2.689
RNF125	ring finger protein 125	-2.697
GGT6	gamma-glutamyltransferase 6	-2.703
KMO	kynurenine 3-monooxygenase	-2.712
LDLR	low density lipoprotein receptor	-2.715
LOC400553	uncharacterized LOC400553	-2.715
MMRN1	multimerin 1	-2.715
ALLC	allantoicase	-2.730
LOC102723701	uncharacterized LOC102723701	-2.739

PAIP2B	poly(A) binding protein interacting protein 2B	-2.739
FOXO1	forkhead box O1	-2.740
AVPR1A	arginine vasopressin receptor 1A	-2.745
KB_68A71		-2.749
KDM8	lysine demethylase 8	-2.751
LIFR	LIF receptor subunit alpha	-2.751
NPY1R	neuropeptide Y receptor Y1	-2.756
SNX29P2	sorting nexin 29 pseudogene 2	-2.756
S100A8	S100 calcium binding protein A8	-2.769
GRAMD4	GRAM domain containing 4	-2.771
MIR17HG	miR-17-92a-1 cluster host gene	-2.773
CD79A	CD79a molecule	-2.774
CA2	carbonic anhydrase 2	-2.775
CYP2C18	cytochrome P450 family 2 subfamily C member 18	-2.782
PDE7B	phosphodiesterase 7B	-2.791
SLC2A9	solute carrier family 2 member 9	-2.800
AL1616685		-2.803
ASS1P1	argininosuccinate synthetase 1 pseudogene 1	-2.805
ASS1P9	argininosuccinate synthetase 1 pseudogene 9	-2.805
IGHV3-30	immunoglobulin heavy variable 3-30	-2.807
XCR1	X-C motif chemokine receptor 1	-2.814
ECM1	extracellular matrix protein 1	-2.818
FNDC5	fibronectin type III domain containing 5	-2.825
ACSL1	acyl-CoA synthetase long chain family member 1	-2.832
STEAP4	STEAP4 metalloreductase	-2.843
ST8SIA6	ST8 alpha-N-acetyl-neuraminide alpha-2,8-sialyltransferase 6	-2.847
AMDHD1	amidohydrolase domain containing 1	-2.848
TNR	tenascin R	-2.855
RP11_404G162		-2.858
ESR1	estrogen receptor 1	-2.859
ARID3C	AT-rich interaction domain 3C	-2.865
MTND4P20	MT-ND4 pseudogene 20	-2.867
HMGCLL1	3-hydroxymethyl-3-methylglutaryl-CoA lyase like 1	-2.879
CSRNP1	cysteine and serine rich nuclear protein 1	-2.884
NIBAN3	niban apoptosis regulator 3	-2.886
SOCS3	suppressor of cytokine signaling 3	-2.894
N4BP2L1	NEDD4 binding protein 2 like 1	-2.898
CD163	CD163 molecule	-2.902
DMGDH	dimethylglycine dehydrogenase	-2.905
ITGAD	integrin subunit alpha D	-2.906
F11-AS1	F11 antisense RNA 1	-2.913
CXCR2P1	C-X-C motif chemokine receptor 2 pseudogene 1	-2.915
FCRL2	Fc receptor like 2	-2.917
DLGAP2	DLG associated protein 2	-2.922
CDH19	cadherin 19	-2.928
SLCO1B1	solute carrier organic anion transporter family member 1B1	-2.931
OAT	ornithine aminotransferase	-2.934
ALB	albumin	-2.940
ST6GAL2	ST6 beta-galactoside alpha-2,6-sialyltransferase 2	-2.945
TTLL2	tubulin tyrosine ligase like 2	-2.945
RP4_568C114		-2.960
NIPAL1	NIPA like domain containing 1	-2.967
PAMR1	peptidase domain containing associated with muscle regeneration 1	-2.967

CLSTN2	calsyntenin 2	-2.968
KIF19	kinesin family member 19	-2.972
LILRA5	leukocyte immunoglobulin like receptor A5	-2.974
ADGRB3	adhesion G protein-coupled receptor B3	-2.975
RP3_453C1214		-2.979
CTD-3080P12.3	uncharacterized LOC101928857	-2.984
IL1RN	interleukin 1 receptor antagonist	-2.999
NTF3	neurotrophin 3	-3.001
GK4P	glycerol kinase 4 pseudogene	-3.006
ASPA	aspartoacylase	-3.007
IL1RAPL2	interleukin 1 receptor accessory protein like 2	-3.024
SLC25A15	solute carrier family 25 member 15	-3.026
GCH1	GTP cyclohydrolase 1	-3.028
CXCL12	C-X-C motif chemokine ligand 12	-3.029
LINC01348	long intergenic non-protein coding RNA 1348	-3.032
LILRB5	leukocyte immunoglobulin like receptor B5	-3.043
CR1	complement C3b/C4b receptor 1 (Knops blood group)	-3.050
SPATA6L	spermatogenesis associated 6 like	-3.062
MT1L	metallothionein 1L, pseudogene	-3.063
PLAC8	placenta associated 8	-3.068
HS1BP3-IT1	HS1BP3 intronic transcript 1	-3.070
TMEM125	transmembrane protein 125	-3.080
EGILA		-3.086
TCIM	transcriptional and immune response regulator	-3.098
PROZ	protein Z, vitamin K dependent plasma glycoprotein	-3.102
AADAT	aminoadipate aminotransferase	-3.115
PPP1R1A	protein phosphatase 1 regulatory inhibitor subunit 1A	-3.116
RETREG1	reticulophagy regulator 1	-3.122
ITLN1	intelectin 1	-3.123
AQP3	aquaporin 3 (Gill blood group)	-3.129
ANKRD55	ankyrin repeat domain 55	-3.133
PTH1R	parathyroid hormone 1 receptor	-3.136
APBA1	amyloid beta precursor protein binding family A member 1	-3.142
ADAMTS13	ADAM metallopeptidase with thrombospondin type 1 motif 13	-3.150
RND1	Rho family GTPase 1	-3.153
B3GAT1	beta-1,3-glucuronyltransferase 1	-3.191
SLCO4C1	solute carrier organic anion transporter family member 4C1	-3.204
LINC01767	long intergenic non-protein coding RNA 1767	-3.205
PADI4	peptidyl arginine deiminase 4	-3.205
MRO	maestro	-3.208
AZGP1P1	AZGP1 pseudogene 1	-3.217
IGKV2-24	immunoglobulin kappa variable 2-24	-3.220
SLCO1B7	solute carrier organic anion transporter family member 1B7 (putative)	-3.226
RP11_90L18		-3.235
CHST4	carbohydrate sulfotransferase 4	-3.236
TMEM82	transmembrane protein 82	-3.244
LCAT	lecithin-cholesterol acyltransferase	-3.246
TDRD15	tudor domain containing 15	-3.247
PLGLA	plasminogen like A (pseudogene)	-3.264
TENM1	teneurin transmembrane protein 1	-3.286
MYOM2	myomesin 2	-3.296
FXYD1	FXYD domain containing ion transport regulator 1	-3.310
LINC01814	long intergenic non-protein coding RNA 1814	-3.310

CXCL2	C-X-C motif chemokine ligand 2	-3.317
MRC1	mannose receptor C-type 1	-3.322
IGLV3-10	immunoglobulin lambda variable 3-10	-3.323
ASS1P11	argininosuccinate synthetase 1 pseudogene 11	-3.333
RP4_564F226		-3.335
IGLV7-43	immunoglobulin lambda variable 7-43	-3.347
FOLH1B	folate hydrolase 1B	-3.349
RP4_669P1016		-3.352
SLC13A2	solute carrier family 13 member 2	-3.368
CNGA1	cyclic nucleotide gated channel subunit alpha 1	-3.373
RP11_71L143		-3.375
DBH	dopamine beta-hydroxylase	-3.377
LINC02362	long intergenic non-protein coding RNA 2362	-3.379
TNFSF11	TNF superfamily member 11	-3.386
MTND5P1	MT-ND5 pseudogene 1	-3.407
RANBP3L	RAN binding protein 3 like	-3.410
NOCT	nocturnin	-3.413
NXF3	nuclear RNA export factor 3	-3.419
SLC7A2	solute carrier family 7 member 2	-3.419
LINC00313	long intergenic non-protein coding RNA 313	-3.430
KCNN2	potassium calcium-activated channel subfamily N member 2	-3.466
AMD1P4	adenosylmethionine decarboxylase 1 pseudogene 4	-3.485
DPF3	double PHD fingers 3	-3.499
PLIN1	perilipin 1	-3.501
MTCYBP21		-3.509
ASS1	argininosuccinate synthase 1	-3.511
FAM151A	family with sequence similarity 151 member A	-3.513
AC1322174		-3.518
LINC00598	long intergenic non-protein coding RNA 598	-3.530
TTC36	tetratricopeptide repeat domain 36	-3.532
CNTFR	ciliary neurotrophic factor receptor	-3.539
SLIT3-AS2	SLIT3 antisense RNA 2	-3.541
RP11_96D16		-3.543
PRSS8	serine protease 8	-3.566
RIPOR3	RIPOR family member 3	-3.598
CYP2C9	cytochrome P450 family 2 subfamily C member 9	-3.604
LOC101928858	uncharacterized LOC101928858	-3.604
TRPC5	transient receptor potential cation channel subfamily C member 5	-3.621
VIPR1	vasoactive intestinal peptide receptor 1	-3.624
IGHM	immunoglobulin heavy constant mu	-3.627
ADGRE1	adhesion G protein-coupled receptor E1	-3.631
SLITRK6	SLIT and NTRK like family member 6	-3.633
IGKV1-9	immunoglobulin kappa variable 1-9	-3.664
GHR	growth hormone receptor	-3.672
SLC51A	solute carrier family 51 subunit alpha	-3.677
BMPER	BMP binding endothelial regulator	-3.692
MEP1B	meprin A subunit beta	-3.703
PDZRN4	PDZ domain containing ring finger 4	-3.710
MT1A	metallothionein 1A	-3.714
AC092053.3		-3.724
CHRM2	cholinergic receptor muscarinic 2	-3.738
MT2A	metallothionein 2A	-3.750
MT1X	metallothionein 1X	-3.753

CAMK2B	calcium/calmodulin dependent protein kinase II beta	-3.762
UNC93A	unc-93 homolog A	-3.767
JCHAIN	joining chain of multimeric IgA and IgM	-3.770
FPR2	formyl peptide receptor 2	-3.784
IGLV4-69	immunoglobulin lambda variable 4-69	-3.801
IGKV2D-29	immunoglobulin kappa variable 2D-29	-3.805
GRHL2	grainyhead like transcription factor 2	-3.807
MOGAT2	monoacylglycerol O-acyltransferase 2	-3.825
DPP10	dipeptidyl peptidase like 10	-3.826
GLT1D1	glycosyltransferase 1 domain containing 1	-3.827
RP11_263G221		-3.836
WNT11	Wnt family member 11	-3.844
GREM2	gremlin 2, DAN family BMP antagonist	-3.845
GPR182	G protein-coupled receptor 182	-3.846
GADD45B	growth arrest and DNA damage inducible beta	-3.847
BMP5	bone morphogenetic protein 5	-3.848
WNT2	Wnt family member 2	-3.868
COX6B1P4	cytochrome c oxidase subunit 6B1 pseudogene 4	-3.892
BCO2	beta-carotene oxygenase 2	-3.907
CTD_2541J132		-3.921
IGKV2-28	immunoglobulin kappa variable 2-28	-3.921
UICLM	up-regulated in colorectal cancer liver metastasis	-3.932
CCBE1	collagen and calcium binding EGF domains 1	-3.939
ANGPTL4	angiopoietin like 4	-3.945
SOCS2-AS1	SOCS2 antisense RNA 1	-3.987
DPT	dermatopontin	-4.007
GCGR	glucagon receptor	-4.013
GNMT	glycine N-methyltransferase	-4.015
IDO2	indoleamine 2,3-dioxygenase 2	-4.022
TIMD4	T cell immunoglobulin and mucin domain containing 4	-4.033
GABRB3	gamma-aminobutyric acid type A receptor subunit beta3	-4.060
GPM6A	glycoprotein M6A	-4.073
RP11_53I62		-4.081
LOC100132813	uncharacterized LOC100132813	-4.099
RP11_196G111		-4.116
DSCAM	DS cell adhesion molecule	-4.123
RP3_342P202		-4.131
IGKV1-17	immunoglobulin kappa variable 1-17	-4.139
FBP1	fructose-bisphosphatase 1	-4.144
IGKV2-30	immunoglobulin kappa variable 2-30	-4.145
CYP3A43	cytochrome P450 family 3 subfamily A member 43	-4.177
ZG16	zymogen granule protein 16	-4.192
CFP	complement factor properdin	-4.228
GABRP	gamma-aminobutyric acid type A receptor subunit pi	-4.233
SLC22A10	solute carrier family 22 member 10	-4.235
OIT3	oncoprotein induced transcript 3	-4.244
ALOX15P1	arachidonate 15-lipoxygenase pseudogene 1	-4.251
HAO2	hydroxyacid oxidase 2	-4.259
PRSS22	serine protease 22	-4.263
LINC01979	long intergenic non-protein coding RNA 1979	-4.278
RSPO3	R-spondin 3	-4.280
ADGRG7	adhesion G protein-coupled receptor G7	-4.326
CETP	cholesteryl ester transfer protein	-4.340

LYVE1	lymphatic vessel endothelial hyaluronan receptor 1	-4.347
AKR1D1	aldo-keto reductase family 1 member D1	-4.444
SLCO1B3	solute carrier organic anion transporter family member 1B3	-4.449
SOCS2	suppressor of cytokine signaling 2	-4.450
CDHR2	cadherin related family member 2	-4.455
LINC00885	long intergenic non-protein coding RNA 885	-4.468
FREM2	FRAS1 related extracellular matrix 2	-4.499
RAB25	RAB25, member RAS oncogene family	-4.505
IGKV1-12	immunoglobulin kappa variable 1-12	-4.512
SFRP5	secreted frizzled related protein 5	-4.523
NRG1	neuregulin 1	-4.567
ESRP1	epithelial splicing regulatory protein 1	-4.597
IGF1	insulin like growth factor 1	-4.668
FAM83F	family with sequence similarity 83 member F	-4.670
MT1E	metallothionein 1E	-4.699
RP11_1035H133		-4.704
MFSD2A	major facilitator superfamily domain containing 2A	-4.728
ANXA8/ANXA8L1	annexin A8	-4.772
ASPG	asparaginase	-4.778
DNASE1L3	deoxyribonuclease 1 like 3	-4.842
IGKV1-6	immunoglobulin kappa variable 1-6	-4.844
IGFALS	insulin like growth factor binding protein acid labile subunit	-4.867
MT1F	metallothionein 1F	-4.871
SLCO1B3-SLCO1B7	SLCO1B3-SLCO1B7 readthrough	-4.898
RPS6KA6	ribosomal protein S6 kinase A6	-4.899
HGFAC	HGF activator	-4.969
COLEC10	collectin subfamily member 10	-4.970
IL1RL1	interleukin 1 receptor like 1	-4.980
HHIP	hedgehog interacting protein	-4.996
CXCL14	C-X-C motif chemokine ligand 14	-5.001
CYP26A1	cytochrome P450 family 26 subfamily A member 1	-5.024
SLC6A19	solute carrier family 6 member 19	-5.030
ADRA1A	adrenoceptor alpha 1A	-5.064
FOSB	FosB proto-oncogene, AP-1 transcription factor subunit	-5.071
CNDP1	carnosine dipeptidase 1	-5.082
IGF2-AS	IGF2 antisense RNA	-5.085
SLC5A1	solute carrier family 5 member 1	-5.242
SLC22A1	solute carrier family 22 member 1	-5.419
LINC01093	long intergenic non-protein coding RNA 1093	-5.436
FCN3	ficolin 3	-5.461
SMUG1P1	single-strand-selective monofunctional uracil-DNA glycosylase 1 pseudogene 1	-5.492
SLC25A47	solute carrier family 25 member 47	-5.506
HAMP	hepcidin antimicrobial peptide	-5.552
COL6A6	collagen type VI alpha 6 chain	-5.598
LPA	lipoprotein(a)	-5.672
FAM180A	family with sequence similarity 180 member A	-5.725
CYP1A2	cytochrome P450 family 1 subfamily A member 2	-5.749
CYP2A6 (includes others)	cytochrome P450 family 2 subfamily A member 6	-5.817
SYT9	synaptotagmin 9	-5.909
NDST3	N-deacetylase and N-sulfotransferase 3	-5.915
STAB2	stabilin 2	-5.947
LINC01488	long intergenic non-protein coding RNA 1488	-5.974

UROC1	urocanate hydratase 1	-6.016
CLEC1B	C-type lectin domain family 1 member B	-6.037
CYP3A7-CYP3A51P	CYP3A7-CYP3A51P readthrough	-6.147
MT1M	metallothionein 1M	-6.221
GLS2	glutaminase 2	-6.338
MARCO	macrophage receptor with collagenous structure	-6.460
SPP2	secreted phosphoprotein 2	-6.470
BMP10	bone morphogenetic protein 10	-6.494
CRHBP	corticotropin releasing hormone binding protein	-6.621
MT1JP	metallothionein 1J, pseudogene	-6.665
RP11_96O204		-6.720
CD5L	CD5 molecule like	-6.723
CLEC4G	C-type lectin domain family 4 member G	-7.007
GDF2	growth differentiation factor 2	-7.496
FCN2	ficolin 2	-7.803
IGF2	insulin like growth factor 2	-7.893
CLEC4M	C-type lectin domain family 4 member M	-8.753

Table S4. Genes differentially expressed in HBV-HCC patients with immune-low subtype.

Gene Symbol	Entrez Gene Name	Fold Change
AKR1B10	aldo-keto reductase family 1 member B10	6.532
AKR1B10P1	aldo-keto reductase family 1 member B10 pseudogene 1	6.497
AKR1B15	aldo-keto reductase family 1 member B15	5.550
MKRN3	makorin ring finger protein 3	5.280
LINC02475	long intergenic non-protein coding RNA 2475	4.813
RP11_284F2110		4.236
CRNDE	colorectal neoplasia differentially expressed	4.142
GSDMC	gasdermin C	4.101
RP11_284F217		4.043
SULT1C2	sulfotransferase family 1C member 2	3.980
AC0072558		3.759
MSL3P1	MSL complex subunit 3 pseudogene 1	3.746
HMMR	hyaluronan mediated motility receptor	3.441
VASH2	vasohibin 2	3.431
LL22NC03-N14H11.1		3.430
CDKN2A	cyclin dependent kinase inhibitor 2A	3.403
RP11_146E134		3.301
CDC25C	cell division cycle 25C	3.228
RBM24	RNA binding motif protein 24	2.901
DUXAP9	double homeobox A pseudogene 9	2.845
CDKN2B-AS1	CDKN2B antisense RNA 1	2.834
CRYBG2	crystallin beta-gamma domain containing 2	2.824
MACIR	macrophage immunometabolism regulator	2.729
ROBO1	roundabout guidance receptor 1	2.672
ITGA2	integrin subunit alpha 2	2.614
DUXAP10	double homeobox A pseudogene 10	2.612
TOB2P1	transducer of ERBB2, 2 pseudogene 1	2.604
DNAJC6	DnaJ heat shock protein family (Hsp40) member C6	2.550
OLFML2B	olfactomedin like 2B	2.514
TP53I3	tumor protein p53 inducible protein 3	2.435
SRXN1	sulfiredoxin 1	2.395
ESCO2	establishment of sister chromatid cohesion N-acetyltransferase 2	2.385
CD109	CD109 molecule	2.346
MAP2	microtubule associated protein 2	2.339
GPX2	glutathione peroxidase 2	2.324
TXNRD1	thioredoxin reductase 1	2.297
RP1_140K85		2.250
ZP3	zona pellucida glycoprotein 3	2.228
SQSTM1	sequestosome 1	2.216
CDKN2B	cyclin dependent kinase inhibitor 2B	2.185
LOC101930100	uncharacterized LOC101930100	2.183
AKR1C3	aldo-keto reductase family 1 member C3	2.152
TKT	transketolase	2.150
C2CD4D-AS1	C2CD4D and THEM5 antisense RNA 1	2.034
SLC26A6	solute carrier family 26 member 6	2.018
ITGA6	integrin subunit alpha 6	1.956
CAP2	cyclase associated actin cytoskeleton regulatory protein 2	1.921
UGT1A6	UDP glucuronosyltransferase family 1 member A6	1.899
HES6	hes family bHLH transcription factor 6	1.831
HSPB1	heat shock protein family B (small) member 1	1.777

TDRKH	tudor and KH domain containing	1.702
ANXA2	annexin A2	1.688
PCSK5	proprotein convertase subtilisin/kexin type 5	1.685
AC0938181		1.631
EPDR1	ependymin related 1	1.624
HEXA-AS1	HEXA antisense RNA 1	1.594
RP11_96D18		1.582
BBLN	bublin coiled coil protein	1.525
S100A10	S100 calcium binding protein A10	1.507
TXN	thioredoxin	1.473
MCTP1	multiple C2 and transmembrane domain containing 1	1.447
FLVCR1	FLVCR heme transporter 1	1.440
CHN1	chimerin 1	1.422
TAGLN2	transgelin 2	1.385
ASPH	aspartate beta-hydroxylase	1.340
HTATIP2	HIV-1 Tat interactive protein 2	1.276
MSTO1	misato mitochondrial distribution and morphology regulator 1	1.249
ATP6V1E2	ATPase H+ transporting V1 subunit E2	1.248
TALDO1	transaldolase 1	1.248
TMEM106C	transmembrane protein 106C	1.245
BBS12	Bardet-Biedl syndrome 12	1.244
TUBG2	tubulin gamma 2	1.196
RHOC	ras homolog family member C	1.111
SKAP2	src kinase associated phosphoprotein 2	1.103
ADAM15	ADAM metallopeptidase domain 15	1.090
MXD3	MAX dimerization protein 3	1.082
AP3S1	adaptor related protein complex 3 subunit sigma 1	1.064
TAF6	TATA-box binding protein associated factor 6	1.053
BSG	basigin (Ok blood group)	1.042
SLC36A1	solute carrier family 36 member 1	1.032
NAGPA	N-acetylglucosamine-1-phosphodiester alpha-N-acetylglucosaminidase	1.028
PLOD3	procollagen-lysine,2-oxoglutarate 5-dioxygenase 3	1.001
DNMBP	dynamin binding protein	-1.000
CCNB1IP1	cyclin B1 interacting protein 1	-1.060
PHLDB2	pleckstrin homology like domain family B member 2	-1.062
PTPN21	protein tyrosine phosphatase non-receptor type 21	-1.141
NR3C2	nuclear receptor subfamily 3 group C member 2	-1.186
TMEM86B	transmembrane protein 86B	-1.199
ARRB1	arrestin beta 1	-1.206
ERLIN1	ER lipid raft associated 1	-1.223
MAPK8IP1	mitogen-activated protein kinase 8 interacting protein 1	-1.228
ATP11C	ATPase phospholipid transporting 11C	-1.233
DIP2C	disco interacting protein 2 homolog C	-1.250
NAV2	neuron navigator 2	-1.275
LOC101927752	uncharacterized LOC101927752	-1.294
GCHFR	GTP cyclohydrolase I feedback regulator	-1.301
ZNF160	zinc finger protein 160	-1.302
FXN	frataxin	-1.309
KLHL15	kelch like family member 15	-1.311
NID1	nidogen 1	-1.314
PRKAG2	protein kinase AMP-activated non-catalytic subunit gamma 2	-1.353
MTARC1	mitochondrial amidoxime reducing component 1	-1.365
FERMT2	FERM domain containing kindlin 2	-1.379

ABHD2	abhydrolase domain containing 2, acylglycerol lipase	-1.380
PRRG4	proline rich and Gla domain 4	-1.381
C21orf91	chromosome 21 open reading frame 91	-1.386
SFXN2	sideroflexin 2	-1.389
DCHS1	dachsous cadherin-related 1	-1.401
TUBE1	tubulin epsilon 1	-1.406
ZRANB1	zinc finger RANBP2-type containing 1	-1.407
P2RY1	purinergic receptor P2Y1	-1.421
ACKR4	atypical chemokine receptor 4	-1.430
CDKN1C	cyclin dependent kinase inhibitor 1C	-1.479
DAPK1	death associated protein kinase 1	-1.495
SYNE1	spectrin repeat containing nuclear envelope protein 1	-1.503
GPHN	gephyrin	-1.514
FAM149A	family with sequence similarity 149 member A	-1.521
ZDHHC1	zinc finger DHHC-type containing 1	-1.525
PXMP2	peroxisomal membrane protein 2	-1.536
C2orf88	chromosome 2 open reading frame 88	-1.541
KLF11	Kruppel like factor 11	-1.558
CDNF	cerebral dopamine neurotrophic factor	-1.575
TIAM1	TIAM Rac1 associated GEF 1	-1.583
ACADS	acyl-CoA dehydrogenase short chain	-1.588
FAM13A	family with sequence similarity 13 member A	-1.594
ARHGEF26	Rho guanine nucleotide exchange factor 26	-1.602
PLPP3	phospholipid phosphatase 3	-1.620
NAAA	N-acylethanolamine acid amidase	-1.702
SMOC1	SPARC related modular calcium binding 1	-1.707
NDRG2	NDRG family member 2	-1.724
MROH8	maestro heat like repeat family member 8	-1.737
HAAO	3-hydroxyanthranilate 3,4-dioxygenase	-1.742
GCDH	glutaryl-CoA dehydrogenase	-1.748
PTPRN2	protein tyrosine phosphatase receptor type N2	-1.752
GPRASP1	G protein-coupled receptor associated sorting protein 1	-1.771
AC1156172		-1.786
OLFML1	olfactomedin like 1	-1.793
LAG3	lymphocyte activating 3	-1.794
PEMT	phosphatidylethanolamine N-methyltransferase	-1.819
PBLD	phenazine biosynthesis like protein domain containing	-1.861
ACACB	acetyl-CoA carboxylase beta	-1.870
N4BP2L1	NEDD4 binding protein 2 like 1	-1.874
PROSER2-AS1	PROSER2 antisense RNA 1	-1.875
IL1RAP	interleukin 1 receptor accessory protein	-1.909
PNMA8B	PNMA family member 8B	-1.924
AQP3	aquaporin 3 (Gill blood group)	-1.936
TMEM26	transmembrane protein 26	-1.975
GSTZ1	glutathione S-transferase zeta 1	-1.980
FOLH1	folate hydrolase 1	-1.984
APBA1	amyloid beta precursor protein binding family A member 1	-1.990
RDH5	retinol dehydrogenase 5	-1.994
ACSM3	acyl-CoA synthetase medium chain family member 3	-2.012
SLC14A1	solute carrier family 14 member 1 (Kidd blood group)	-2.021
DLEC1	DLEC1 cilia and flagella associated protein	-2.027
SULT1C4	sulfotransferase family 1C member 4	-2.035
CHRNE	cholinergic receptor nicotinic epsilon subunit	-2.044

SMAD6	SMAD family member 6	-2.044
RP11_93B149		-2.057
OAT	ornithine aminotransferase	-2.066
DAAM2	dishevelled associated activator of morphogenesis 2	-2.069
SH3YL1	SH3 and SYLF domain containing 1	-2.087
CFAP251	cilia and flagella associated protein 251	-2.104
LINC00261	long intergenic non-protein coding RNA 261	-2.110
SCD5	stearoyl-CoA desaturase 5	-2.126
LOC102723701	uncharacterized LOC102723701	-2.172
PRUNE2	prune homolog 2 with BCH domain	-2.172
SYNGR1	synaptogyrin 1	-2.184
TBXA2R	thromboxane A2 receptor	-2.189
IGFBP3	insulin like growth factor binding protein 3	-2.190
RBMS3	RNA binding motif single stranded interacting protein 3	-2.193
CTC_526N191		-2.197
CTH	cystathionine gamma-lyase	-2.207
LIPG	lipase G, endothelial type	-2.222
DBH-AS1	DBH antisense RNA 1	-2.261
LOC100505918	uncharacterized LOC100505918	-2.261
TMEM220	transmembrane protein 220	-2.262
LCAT	lecithin-cholesterol acyltransferase	-2.266
RASSF9	Ras association domain family member 9	-2.268
NIPAL1	NIPA like domain containing 1	-2.282
BCHE	butyrylcholinesterase	-2.283
DLG2	discs large MAGUK scaffold protein 2	-2.289
FEZ1	fasciculation and elongation protein zeta 1	-2.298
TECTA	tectorin alpha	-2.303
TSPOAP1	TSPO associated protein 1	-2.316
KLHL3	kelch like family member 3	-2.328
LRAT	lecithin retinol acyltransferase	-2.328
PANK1	pantothenate kinase 1	-2.334
SNX29P2	sorting nexin 29 pseudogene 2	-2.337
CPEB3	cytoplasmic polyadenylation element binding protein 3	-2.340
SMPD3	sphingomyelin phosphodiesterase 3	-2.359
CCDC3	coiled-coil domain containing 3	-2.365
EPB41L4A	erythrocyte membrane protein band 4.1 like 4A	-2.385
CIDEB	cell death inducing DFFA like effector b	-2.393
PPARGC1A	PPARG coactivator 1 alpha	-2.395
TLL1	tolloid like 1	-2.395
CA3	carbonic anhydrase 3	-2.403
RP11_42O153		-2.435
ITGA9	integrin subunit alpha 9	-2.436
RP11_236L142		-2.455
ADGRE1	adhesion G protein-coupled receptor E1	-2.460
MMRN1	multimerin 1	-2.460
RTL5	retrotransposon Gag like 5	-2.460
DPF3	double PHD fingers 3	-2.491
FAT4	FAT atypical cadherin 4	-2.494
KCNJ10	potassium inwardly rectifying channel subfamily J member 10	-2.505
MYO10	myosin X	-2.505
ATP1B2	ATPase Na+/K+ transporting subunit beta 2	-2.513
LRRC7	leucine rich repeat containing 7	-2.562
LTK	leukocyte receptor tyrosine kinase	-2.566

GRAMD1C	GRAM domain containing 1C	-2.573
IFITM10	interferon induced transmembrane protein 10	-2.579
MRO	maestro	-2.657
CD1D	CD1d molecule	-2.681
MYOM2	myomesin 2	-2.683
SEMA6D	semaphorin 6D	-2.720
SERPINA5	serpin family A member 5	-2.731
GNA14	G protein subunit alpha 14	-2.735
PBX1	PBX homeobox 1	-2.762
PAMR1	peptidase domain containing associated with muscle regeneration 1	-2.783
ERRFI1	ERBB receptor feedback inhibitor 1	-2.797
ADIRF	adipogenesis regulatory factor	-2.799
CNTN4	contactin 4	-2.801
AADAT	aminoadipate aminotransferase	-2.812
RP11_71L143		-2.830
DOCK8-AS1	DOCK8 antisense RNA 1	-2.851
KAZN	kazrin, periplakin interacting protein	-2.856
OLFML3	olfactomedin like 3	-2.857
LINC01814	long intergenic non-protein coding RNA 1814	-2.858
CROCC2	ciliary rootlet coiled-coil, rootletin family member 2	-2.861
ACADSB	acyl-CoA dehydrogenase short/branched chain	-2.883
TPPP2	tubulin polymerization promoting protein family member 2	-2.892
RP11_96D16		-2.898
LINC01767	long intergenic non-protein coding RNA 1767	-2.905
KBTBD11	kelch repeat and BTB domain containing 11	-2.910
PDK4	pyruvate dehydrogenase kinase 4	-2.942
EVC	EvC ciliary complex subunit 1	-2.943
ANGPTL6	angiopoietin like 6	-2.973
KIF19	kinesin family member 19	-2.976
KMO	kynurenine 3-monooxygenase	-2.979
HUNK	hormonally up-regulated Neu-associated kinase	-2.981
DACH1	dachshund family transcription factor 1	-2.995
SOCS2	suppressor of cytokine signaling 2	-2.995
MTND4P20	MT-ND4 pseudogene 20	-2.997
CFP	complement factor properdin	-3.026
FUT3	fucosyltransferase 3 (Lewis blood group)	-3.033
SLIT3-AS2	SLIT3 antisense RNA 2	-3.038
LINC00365	long intergenic non-protein coding RNA 365	-3.041
LYVE1	lymphatic vessel endothelial hyaluronan receptor 1	-3.042
SULT1E1	sulfotransferase family 1E member 1	-3.052
XDH	xanthine dehydrogenase	-3.057
C1QTNF7	C1q and TNF related 7	-3.060
PROM2	prominin 2	-3.069
TRPV4	transient receptor potential cation channel subfamily V member 4	-3.092
PRRT1B	proline rich transmembrane protein 1B	-3.095
COLEC11	collectin subfamily member 11	-3.124
ECM1	extracellular matrix protein 1	-3.149
LIFR	LIF receptor subunit alpha	-3.154
ANKRD55	ankyrin repeat domain 55	-3.164
ANK3	ankyrin 3	-3.169
KDM8	lysine demethylase 8	-3.170
ADRA2B	adrenoceptor alpha 2B	-3.173
INMT	indolethylamine N-methyltransferase	-3.209

SHROOM2	shroom family member 2	-3.226
RNF150	ring finger protein 150	-3.228
COX6B1P4	cytochrome c oxidase subunit 6B1 pseudogene 4	-3.232
IDO2	indoleamine 2,3-dioxygenase 2	-3.246
PTN	pleiotrophin	-3.251
DBH	dopamine beta-hydroxylase	-3.258
MTCYBP21		-3.271
RAB36	RAB36, member RAS oncogene family	-3.286
BRINP1	BMP/retinoic acid inducible neural specific 1	-3.292
GNMT	glycine N-methyltransferase	-3.328
CETP	cholesteryl ester transfer protein	-3.339
EHD3	EH domain containing 3	-3.339
HGF	hepatocyte growth factor	-3.345
PLAC8	placenta associated 8	-3.373
RP11_263G221		-3.382
ADGRG7	adhesion G protein-coupled receptor G7	-3.388
ALLC	allantoicase	-3.396
SGCA	sarcoglycan alpha	-3.421
ALKAL2	ALK and LTK ligand 2	-3.433
PITPNM3	PITPNM family member 3	-3.451
POU2AF1	POU class 2 homeobox associating factor 1	-3.468
UNC93A	unc-93 homolog A	-3.489
TACSTD2	tumor associated calcium signal transducer 2	-3.504
TCF21	transcription factor 21	-3.520
ESR1	estrogen receptor 1	-3.522
LINC01348	long intergenic non-protein coding RNA 1348	-3.559
AL1616685		-3.567
PLPP4	phospholipid phosphatase 4	-3.573
CLSTN2	calsyntenin 2	-3.587
RANBP3L	RAN binding protein 3 like	-3.587
LRRN3	leucine rich repeat neuronal 3	-3.609
CYP2C9	cytochrome P450 family 2 subfamily C member 9	-3.612
NIBAN3	niban apoptosis regulator 3	-3.630
SLC16A9	solute carrier family 16 member 9	-3.653
NXF3	nuclear RNA export factor 3	-3.667
MZB1	marginal zone B and B1 cell specific protein	-3.676
RIC3	RIC3 acetylcholine receptor chaperone	-3.677
ATP13A4	ATPase 13A4	-3.684
ATOH8	atonal bHLH transcription factor 8	-3.691
SLC13A2	solute carrier family 13 member 2	-3.699
ASPA	aspartoacylase	-3.733
GPR182	G protein-coupled receptor 182	-3.741
RP4_568C114		-3.769
SELP	selectin P	-3.770
GNAO1	G protein subunit alpha o1	-3.790
PPP1R1A	protein phosphatase 1 regulatory inhibitor subunit 1A	-3.790
LGR6	leucine rich repeat containing G protein-coupled receptor 6	-3.806
PTGIS	prostaglandin I2 synthase	-3.806
PHACTR3	phosphatase and actin regulator 3	-3.812
DYNLT5	dynein light chain Tctex-type family member 5	-3.823
TMEM132E	transmembrane protein 132E	-3.830
CYP39A1	cytochrome P450 family 39 subfamily A member 1	-3.847
DNASE1L3	deoxyribonuclease 1 like 3	-3.882

P2RX3	purinergic receptor P2X 3	-3.882
ADAMTS13	ADAM metallopeptidase with thrombospondin type 1 motif 13	-3.929
TTC36	tetratricopeptide repeat domain 36	-3.935
RIPOR3	RIPOR family member 3	-3.968
CLIC6	chloride intracellular channel 6	-3.996
MFSD2A	major facilitator superfamily domain containing 2A	-4.007
CXCL12	C-X-C motif chemokine ligand 12	-4.070
SRPX	sushi repeat containing protein X-linked	-4.075
CFAP46	cilia and flagella associated protein 46	-4.114
VIPR1-AS1	VIPR1 antisense RNA 1	-4.118
OIT3	oncoprotein induced transcript 3	-4.130
KCNJ3	potassium inwardly rectifying channel subfamily J member 3	-4.135
UICLM	up-regulated in colorectal cancer liver metastasis	-4.196
RP3_342P202		-4.204
AJAP1	adherens junctions associated protein 1	-4.212
TNR	tenascin R	-4.222
KCNK17	potassium two pore domain channel subfamily K member 17	-4.224
LRRN1	leucine rich repeat neuronal 1	-4.271
DLGAP2	DLG associated protein 2	-4.273
DSCAM	DS cell adhesion molecule	-4.275
RP11_39H32		-4.276
CDHR2	cadherin related family member 2	-4.290
FOLH1B	folate hydrolase 1B	-4.299
SLITRK6	SLIT and NTRK like family member 6	-4.313
PLXNA4	plexin A4	-4.320
ITGB8	integrin subunit beta 8	-4.352
ADGRB3	adhesion G protein-coupled receptor B3	-4.363
WNT2	Wnt family member 2	-4.382
PTH1R	parathyroid hormone 1 receptor	-4.394
IGLV1-51	immunoglobulin lambda variable 1-51	-4.412
LINC02593	long intergenic non-protein coding RNA 2593	-4.419
SPSB4	splA/ryanodine receptor domain and SOCS box containing 4	-4.504
HMGCLL1	3-hydroxymethyl-3-methylglutaryl-CoA lyase like 1	-4.517
CTNNA3	catenin alpha 3	-4.531
TTLL2	tubulin tyrosine ligase like 2	-4.560
NRG3	neuregulin 3	-4.583
RSPO3	R-spondin 3	-4.603
SLC22A10	solute carrier family 22 member 10	-4.625
TIMD4	T cell immunoglobulin and mucin domain containing 4	-4.639
BEND4	BEN domain containing 4	-4.640
IL13RA2	interleukin 13 receptor subunit alpha 2	-4.646
FAM30A	family with sequence similarity 30 member A	-4.672
LINC00885	long intergenic non-protein coding RNA 885	-4.679
AVPR1A	arginine vasopressin receptor 1A	-4.718
CNTFR	ciliary neurotrophic factor receptor	-4.720
CNDP1	carnosine dipeptidase 1	-4.768
FREM1	FRAS1 related extracellular matrix 1	-4.769
SMUG1P1	single-strand-selective monofunctional uracil-DNA glycosylase 1 pseudogene 1	-4.800
DSCAML1	DS cell adhesion molecule like 1	-4.803
ST6GAL2	ST6 beta-galactoside alpha-2,6-sialyltransferase 2	-4.824
CYP26A1	cytochrome P450 family 26 subfamily A member 1	-4.828
IGLV2-8	immunoglobulin lambda variable 2-8	-4.851
NTS	neurotensin	-4.865

LINC01979	long intergenic non-protein coding RNA 1979	-4.888
KCNN2	potassium calcium-activated channel subfamily N member 2	-4.908
PDX1	pancreatic and duodenal homeobox 1	-4.934
FREM2	FRAS1 related extracellular matrix 2	-4.946
FXYD2	FXYD domain containing ion transport regulator 2	-4.962
NPBWR1	neuropeptides B and W receptor 1	-4.994
NUDT10	nudix hydrolase 10	-5.014
CHRM2	cholinergic receptor muscarinic 2	-5.068
HAO2	hydroxyacid oxidase 2	-5.089
B3GAT1	beta-1,3-glucuronyltransferase 1	-5.091
AC1322174		-5.159
PWWP3B	PWWP domain containing 3B	-5.193
IGKV2-24	immunoglobulin kappa variable 2-24	-5.209
VIPR1	vasoactive intestinal peptide receptor 1	-5.227
CAPN13	calpain 13	-5.238
SPP2	secreted phosphoprotein 2	-5.238
HHIP	hedgehog interacting protein	-5.246
ANXA8/ANXA8L1	annexin A8	-5.247
CLDN10	claudin 10	-5.254
RPS6KA6	ribosomal protein S6 kinase A6	-5.315
B3GALT5	beta-1,3-galactosyltransferase 5	-5.378
CD5L	CD5 molecule like	-5.378
IGHA1	immunoglobulin heavy constant alpha 1	-5.392
WNK2	WNK lysine deficient protein kinase 2	-5.403
ASPG	asparaginase	-5.438
IGHV5-51	immunoglobulin heavy variable 5-51	-5.481
MUC6	mucin 6, oligomeric mucus/gel-forming	-5.543
FGFR2	fibroblast growth factor receptor 2	-5.560
CCBE1	collagen and calcium binding EGF domains 1	-5.567
IGFALS	insulin like growth factor binding protein acid labile subunit	-5.644
CFTR	CF transmembrane conductance regulator	-5.675
FXYD1	FXYD domain containing ion transport regulator 1	-5.689
COLEC10	collectin subfamily member 10	-5.727
CRHBP	corticotropin releasing hormone binding protein	-5.743
IGHV1-24	immunoglobulin heavy variable 1-24	-5.744
PRSS8	serine protease 8	-5.795
SLC25A47	solute carrier family 25 member 47	-5.875
COL6A6	collagen type VI alpha 6 chain	-5.895
IGF2-AS	IGF2 antisense RNA	-5.906
DPP10	dipeptidyl peptidase like 10	-5.913
CXCL14	C-X-C motif chemokine ligand 14	-5.922
MARCO	macrophage receptor with collagenous structure	-6.091
BICDL2	BICD family like cargo adaptor 2	-6.120
BMP10	bone morphogenetic protein 10	-6.163
FCN3	ficolin 3	-6.169
CYP1A2	cytochrome P450 family 1 subfamily A member 2	-6.272
ERVE-1	endogenous retrovirus group E member 1	-6.281
LINC01093	long intergenic non-protein coding RNA 1093	-6.307
BMPER	BMP binding endothelial regulator	-6.352
SYT9	synaptotagmin 9	-6.377
CHST4	carbohydrate sulfotransferase 4	-6.384
FAM83F	family with sequence similarity 83 member F	-6.390
CLEC4G	C-type lectin domain family 4 member G	-6.430

RAB25	RAB25, member RAS oncogene family	-6.515
NDST3	N-deacetylase and N-sulfotransferase 3	-6.535
CLEC1B	C-type lectin domain family 1 member B	-6.625
GGT6	gamma-glutamyltransferase 6	-6.727
STAB2	stabilin 2	-6.858
HAMP	hepcidin antimicrobial peptide	-6.897
FCN2	ficolin 2	-6.909
GRHL2	grainyhead like transcription factor 2	-7.243
ESRP1	epithelial splicing regulatory protein 1	-7.268
LINC01488	long intergenic non-protein coding RNA 1488	-7.486
CLEC4M	C-type lectin domain family 4 member M	-7.569
SLC5A1	solute carrier family 5 member 1	-7.623
GDF2	growth differentiation factor 2	-7.798
SFRP5	secreted frizzled related protein 5	-8.404
IGF2	insulin like growth factor 2	-8.766

Table S5. Ingenuity pathway analysis of the unpaired comparison performed between tumors with immune-high and immune-low subtypes.

**Ingenuity Canonical Pathways**	**-log(p-value)**	**z-score**
Neuroinflammation signaling pathway	4.73	-2.694
PD-1, PD-L1 cancer immunotherapy pathway	3.36	-0.277
WNT/Ca+ pathway	2.87	1.667
IL-10 signaling	2.82	1.606
TREM1 signaling	2.82	-2.111
Antigen presentation pathway	2.81	-
Pathogen induced cytokine storm signaling pathway	2.48	-2.874
Role of pattern recognition receptors in recognition of bacteria and viruses	2.38	-2.111
Th1 pathway	2.33	-1.807
Th1 and Th2 activation pathway	2.28	-
Dermatan sulfate biosynthesis	2.09	2.333
Amyloid processing	2.06	-
L-carnitine biosynthesis	2.01	-
N-acetylglucosamine degradation I	2.01	-
Role of macrophages, fibroblasts and endothelial cells in rheumatoid arthritis	1.96	-
Regulation of the epithelial mesenchymal transition in development pathway	1.93	1.414
Pathogenesis of multiple sclerosis	1.90	-
IL-17A signaling in airway cells	1.83	0.000
Colorectal cancer metastasis signaling	1.82	0.447
CLEAR signaling pathway	1.82	0.600
